# Loss of ATOH1 in Pit Cell Drives Stemness and Progression of Gastric Adenocarcinoma by Activating AKT/mTOR Signaling through GAS1

**DOI:** 10.1002/advs.202301977

**Published:** 2023-10-12

**Authors:** Qing Zhong, Hua‐Gen Wang, Ji‐Hong Yang, Ru‐Hong Tu, An‐Yao Li, Gui‐Rong Zeng, Qiao‐Ling Zheng, Zhi‐ Yu Liu, Zhi‐Xin Shang‐Guan, Xiao‐ Bo Huang, Qiang Huang, Yi‐Fan Li, Hua‐Long Zheng, Guang‐Tan Lin, Ze‐Ning Huang, Kai‐Xiang Xu, Wen‐Wu Qiu, Mei‐Chen Jiang, Ya‐Jun Zhao, Jian‐Xian Lin, Zhi‐Hong Huang, Jing‐Min Huang, Ping Li, Jian‐Wei Xie, Chao‐Hui Zheng, Qi‐Yue Chen, Chang‐Ming Huang

**Affiliations:** ^1^ Department of Gastric Surgery Fujian Medical University Union Hospital Fuzhou 350001 P. R. China; ^2^ Department of General Surgery Fujian Medical University Union Hospital Fuzhou 350001 P. R. China; ^3^ Key Laboratory of Ministry of Education of Gastrointestinal Cancer Fujian Medical University Fuzhou 350122 P. R. China; ^4^ BoYu Intelligent Health Innovation Laboratory Hangzhou 311100 P. R. China; ^5^ College of Pharmaceutical Sciences Zhejiang University Hangzhou 310058 P. R. China; ^6^ Department of Pathology Fujian Medical University Union Hospital Fuzhou 350001 P. R. China; ^7^ Diagnostic Pathology Center Fujian Medical University Fuzhou 350001 P. R. China; ^8^ Department of Gastrointestinal Surgery The First Affiliated Hospital of the University of Science and Technology of China Division of Life Sciences and Medicine University of Science and Technology of China Hefei 230001 P. R. China; ^9^ Public Technology Service Center Fujian Medical University Fuzhou 350122 P. R. China; ^10^ Department of General Surgery Qinghai Provincial People's Hospital Xining 810000 P. R. China

**Keywords:** ATOH1, GAS1, gastric adenocarcinoma, mouse model, stemness

## Abstract

Gastric cancer stem cells (GCSCs) are self‐renewing tumor cells that govern chemoresistance in gastric adenocarcinoma (GAC), whereas their regulatory mechanisms remain elusive. Here, the study aims to elucidate the role of *ATOH1* in the maintenance of GCSCs. The preclinical model and GAC sample analysis indicate that *ATOH1* deficiency is correlated with poor GAC prognosis and chemoresistance. ScRNA‐seq reveals that *ATOH1* is downregulated in the pit cells of GAC compared with those in paracarcinoma samples. Lineage tracing reveals that *Atoh1* deletion strongly confers pit cell stemness. *ATOH1* depletion significantly accelerates cancer stemness and chemoresistance in *Tff1‐CreERT2; Rosa26^Tdtomato^* and *Tff1‐CreERT2; Apc^fl/fl^; p53^fl/fl^* (*TcPP*) mouse models and organoids. *ATOH1* deficiency downregulates growth arrest‐specific protein 1 (*GAS1*) by suppressing *GAS1* promoter transcription. *GAS1* forms a complex with *RET*, which inhibits Tyr1062 phosphorylation, and consequently activates the *RET*/*AKT*/*mTOR* signaling pathway by *ATOH1* deficiency. Combining chemotherapy with drugs targeting *AKT*/*mTOR* signaling can overcome *ATOH1* deficiency‐induced chemoresistance. Moreover, it is confirmed that abnormal DNA hypermethylation induces *ATOH1* deficiency. Taken together, the results demonstrate that *ATOH1* loss promotes cancer stemness through the *ATOH1*/*GAS1*/*RET*/*AKT*/*mTOR* signaling pathway in GAC, thus providing a potential therapeutic strategy for *AKT*/*mTOR* inhibitors in GAC patients with *ATOH1* deficiency.

## Graphical abstract

In this study, through survival analysis in different samples and multifarious experiments, an important mechanism is identified in the stemness of gastric adenocarcinoma: the deficiency of ATOH1‐regulated by its DNMT1 level blocks GAS1 promoter transcription, further activating the RET/AKT/mTOR pathway and promoting CSC‐like qualities and chemotherapy resistance, and hence presents a significant association with the poor prognosis.

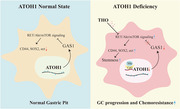

## 1 Introduction

Gastric adenocarcinoma (GAC) is the fifth most common cancer worldwide and the third leading cause of cancer‐related deaths.^[^ ^1^ ^]^ Chemotherapy and tumor recurrences are persistent and unresolved problems associted with GAC treatment.^[^ ^2^ ^]^ Gastric cancer stem cells (GCSCs) are a small population of self‐renewing tumor cells isolated from GAC.^[^ ^3^ ^]^ As GCSCs have inherent stem cell‐like properties, they play vital roles in tumor progression and therapeutic resistance. The ability of CSCs to adopt a quiescent state has emerged as an important driver of drug resistance.^[^ ^4^ ^]^ Unfortunately, the low efficacy of conventional 5‐FU‐based chemotherapy against GCSCs often leads to treatment failure.^[^ ^5^ ^]^ Elucidating the regulatory mechanisms of GCSCs may facilitate the development of novel targeted strategies to eliminate these cells and improve the prognosis of GAC.

Atonal basic helix‐loop‐helix transcription factor 1 (*ATOH1*) is a member of the basic helix‐loop‐helix (bHLH) family of transcription factors that are involved in various developmental processes.^[^ ^6^ ^]^ *ATOH1* specifies and regulates the skin mechanosensory cells and develops the auditory system in the inner ear.^[^ ^7^ ^]^ To the best of our knowledge, the role of *ATOH1* in gastric epithelial development has not been reported. In addition, although certain studies have demonstrated that *ATOH1* participates in carcinogenesis,^[^ ^8^ ^]^ its specific role and mechanism in this process in GAC still need to be clarified. Therefore, this study aimed to investigate the effects of *ATOH1* on the GCSC phenotype and chemotherapy resistance in GAC.

To determine the roles of *ATOH1* in GAC, we established a stomach‐specific *Atoh1* transgenic mouse model and evaluated *Atoh1* deletion as a risk factor for GAC progression. Stomach‐specific *Atoh1* deletion promotes stemness and chemoresistance of gastric epithelial cells. Moreover, *ATOH1* downregulation results in poor GAC prognosis. *ATOH1* inhibits stemness and chemoresistance in the GAC by activating growth arrest‐specific protein 1 (*GAS1)* transcription and suppressing the *RET*/*AKT*/*mTOR* signaling pathway. Therefore, *ATOH1* is a promising therapeutic target for the treatment of GAC.

## 2 Results

### 2.1 *ATOH1* is Downregulated in Chemoresistant GAC Tumors and GAC Pit Cells

**Figure 1:**
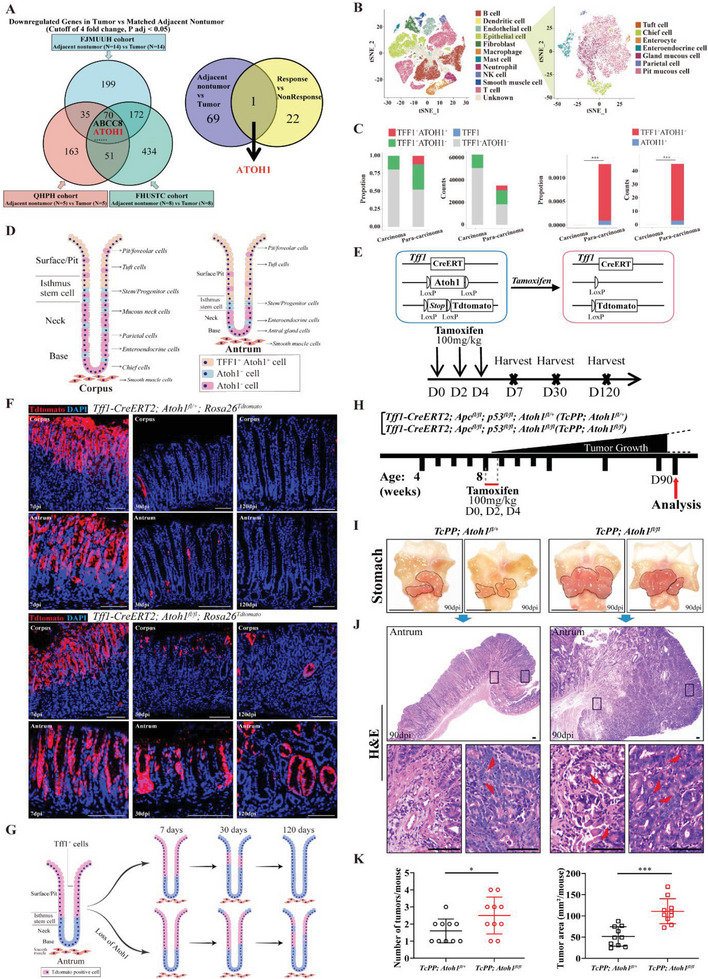
*ATOH1* loss increases spontaneous tumorigenesis in a mouse model. A) Flowchart showing a screening of candidate genes orchestrating human GAC stemness. Venn diagram (left) showing overlap of downregulated genes in human GAC compared with corresponding adjacent non‐tumor tissues from FJMUUH, QHPH, and FHUSTC cohorts. Venn diagram (right) showing overlap of downregulated genes in GAC versus adjacent non‐tumor tissues and chemoresistant versus chemosensitive tumors. B) scRNA‐seq analysis of integrated cells isolated from eight GAC samples and eleven paracarcinoma samples based on notable cell type markers (Carcinoma cohort: n = 8, Paracarcinoma cohort: *n* = 11). C) Histogram indicating *ATOH1* downregulation in *TFF1* ^+^ pit cells isolated from GAC samples compared to paracarcinoma samples. D) Schematic diagram of *Tff1* and *Atoh1* expression in mouse stomach. E) Schematic diagram of *Tff1‐CreERT2; Atoh1^fl/fl^; Rosa26^Tdtomato^* mouse generation. F) Representative images of *Tff1‐CreERT2; Atoh1^fl/fl^; Rosa26^Tdtomato^*, and *Tff1‐CreERT2; Rosa26^Tdtomato^* mouse lineage tracing at 7, 30, and 120 dpi (scale bars = 100 µm). G) Working model for roles of *Atoh1* in gastric epithelium maintenance. H) Experimental design for tamoxifen administration and analysis. I) Representative macroscopic views of stomachs of *Tff1‐CreERT2; Apc^fl/fl^; p53^fl/fl^; Atoh1^fl/+^* (*TcPP; Atoh1^fl/+^*) and *Tff1‐CreERT2; Apc^fl/fl^; p53^fl/fl^; Atoh1^fl/fl^* (*TcPP; Atoh1^fl/fl^*) mice collected 90 days after tamoxifen administration. Tumors are indicated by red arrows (scale bars = 1 cm). J) Representative H&E staining of stomachs of *TcPP; Atoh1^fl/+^* and *TcPP; Atoh1^fl/fl^* mice collected 90 days after tamoxifen administration (scale bars = 100 µm). K) Total numbers (left) and areas (right) of mouse tumors harvested from *TcPP; Atoh1^fl/+^* and *TcPP; Atoh1^fl/fl^* mice (*n* = 10 per cohort) at 90 days after tamoxifen administration. Data are represented as the mean ± SD and analyzed by Student's *t*‐test. *P <0.05, ***P <0.001 for groups connected by horizontal lines. Data with *p*‐value < 0.05 were considered statistically significant.

Resistance to chemotherapy is a manifestation of GAC stemness.^[^ ^9^ ^]^ We, therefore, sought genes that were preferentially downregulated in GAC (vs adjacent gastric tissues) and chemoresistant GAC (vs chemosensitive GAC). We identified the expression profiles (Figure S1A, Supporting Information) of dysregulated genes in three GAC versus adjacent gastric non‐tumor cohorts from Fujian Medical University Union Hospital (FJMUUH), First Affiliated Hospital of the University of Science and Technology of China (FHUSTC), and Qinghai Provincial People's Hospital (QHPH). We found 476, 727, and 319 downregulated genes with logFC < −2 and adjusted *P* < 0.05 in the FJMUUH, FHUSTC, and QHPH cohorts, respectively. Venn diagrams revealed 70 downregulated genes common to all three cohorts (**Figure** **1**A). Twenty‐three downregulated genes were detected in the chemoresistant and chemosensitive GAC cases from FJMUUH (Figure S1B,C, Supporting Information). These two groups overlapped only in *ATOH1* (Figure 1A). *ATOH1* downregulation was detected in chemoresistant cells (Figure S1D, Supporting Information). *ATOH1* mRNA expression was significantly decreased in GAC versus adjacent normal tissues from the FJMUUH cohort (Figure S1E, Supporting Information). *ATOH1* protein levels were significantly lower in 147 primary tumor samples than in adjacent noncancerous tissues from FJMUUH patients (Figure S1F, Supporting Information). Four out of ten wild‐type C57BL6 mice developed GAC 12 months after MNU induction (Figure S1G, Supporting Information). The proportions of *Atoh1* ^+^ cells were significantly lower in MNU‐induced mouse tumors (corpus: 53.5 ± 3.6% vs 8.7 ± 2.8%, *P* < 0.001; antrum: 58.4 ± 4.8% vs 11.5 ± 3.1%, *P* < 0.001) than the normal gastric tissues (Figure S1G, Supporting Information).

We performed single‐cell transcriptome sequencing (scRNA‐seq) on GAC and paracarcinoma samples from the present and a previously published study (Table S4, Supporting Information) (Figure 1B; Figure S2A–C, Supporting Information).^[^ ^10^ ^]^ Differential gene expression analysis identified several markers associated with the cultured gastric epithelium and their expression in gastric epithelial cells are shown by t‐distributed stochastic neighbor embedding (tSNE), such as *PGAC*, *MUC5AC*, and *TFF1* (Figure 1B; Figure S2D, Supporting Information).^[^ ^11^ ^]^ Moreover, *ATOH1* is barely expressed in the *TFF1* ^+^ epithelial (pit) cells of the GAC samples, but not in the paracarcinoma samples (Figure 1C; Figure S2E,F, Supporting Information). These findings demonstrated that the number of *ATOH1* ^+^ gastric epithelial cells decreased after oncogenic stimulation. Furthermore, the loss of *Atoh1* in pit cells shaped cellular interactions and the tumor microenvironment (Figure S3A–D, Supporting Information).

### 2.2 *ATOH1* Deletion in Mouse Stomach Pit Cells Promotes Cancer Stemness and Aggressiveness

Endogenous *TFF1* was expressed in the pit regions of the gastric glands in the corpus and antrum and co‐localized with *Atoh1* in pit cells (Figure S4A–C, Supporting Information).^[^ ^11^, ^12^ ^]^ We generated *Tff1‐CreERT2; Rosa26^Tdtomato^* mice and confirmed that the stomachs of *Tff1* ^+^ lineage mice contained *Atoh1* ^+^ cells, whereas the *Tff1* ^+^ lineage was not detected in the small intestine or colon (Figure 1D; Figure S4C,D, Supporting Information). Tamoxifen administration silenced the *Atoh1* protein in *Tff1* ^+^ cells in the gastric epithelia of *Tff1‐CreERT2; Atoh1^fl/fl^; Rosa26^Tdtomato^* mice (Figure 1E; Figure S4E,F, Supporting Information). Lineage tracing showed that *Tff1* ^+^ cells proliferated in the absence of *Atoh1* (Figure 1F,G). Persistent *Apc* and *p53* ablation led to gastric tumorigenesis 90 days after tamoxifen induction in *Tff1‐CreERT2; Apc^fl/fl^; p53^fl/fl^* (*TcPP*) mice (Figure 1H–J). We hypothesized that *Tff1‐CreERT2* transgene‐mediated *Atoh1* ablation would enhance this effect. Hence, we administered tamoxifen to *TcPP* and *Tff1‐CreERT2; Apc^fl/fl^; p53^fl/fl^; Atoh1^fl/fl^* (*TcPP; Atoh1^fl/fl^*) mice harboring the “floxed” *ATOH1* allele (Figure S4G, Supporting Information). We observed significant increases in tumor burden and number in *Atoh1^fl/fl^* cohort mice 90 days after tamoxifen induction (Figure 1I–K, Supporting Information).

### 2.3 *ATOH1* Inhibits Cancer Stemness In Vivo and In Vitro

**Figure 2:**
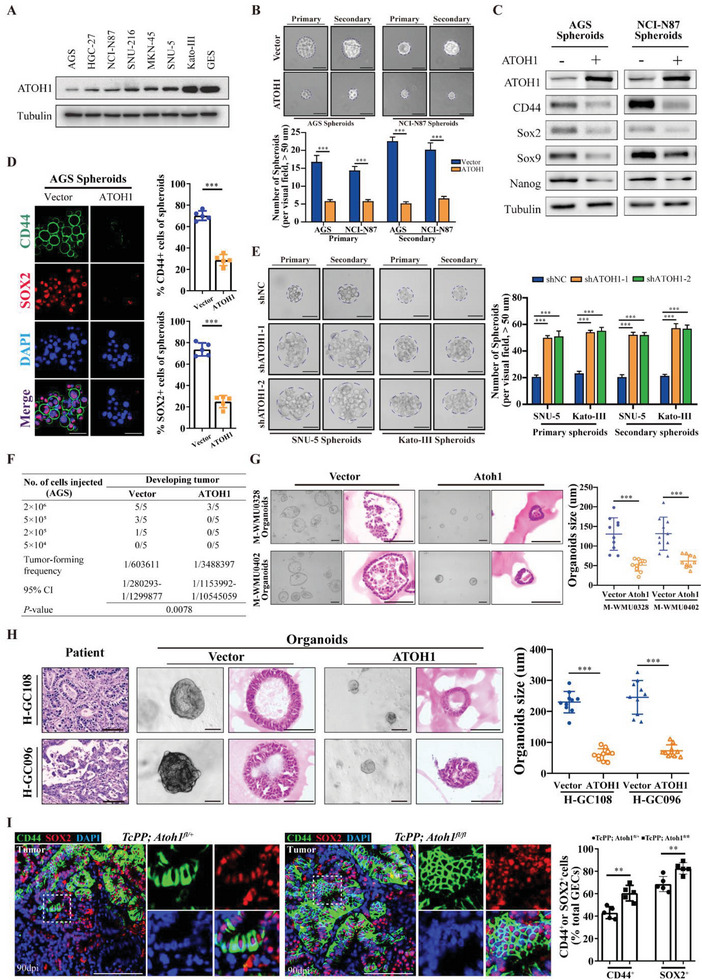
*ATOH1* expression is correlated with CSC phenotype in GAC cells. A) Western blot of *ATOH1* in immortalized gastric epithelial cells and GAC cell panel. B) Spheroid formation by AGS and NCI‐N87 cells transfected with *ATOH1* or vector (scale bars = 50 µm, *n* = 5). C) Western blot of CSC and self‐renewal markers in AGS and NCI‐N87 spheroids transfected with *ATOH1* or vector. D) Quantification and immunofluorescence images of *CD44* and *SOX2* in AGS spheroids transfected with *ATOH1* or vector (scale bars = 50 µm). E) Spheroid formation transfected with sh*ATOH1* or sh*NC* (scale bars = 50 µm, *n* = 5). F) AGS cells with or without *ATOH1* overexpression were serially diluted and subcutaneously xenografted into BALB/c nude mice. Number of cells injected and tumor formation frequency on day 28 are shown. G) Effects of *ATOH1* overexpression on MNU mouse‐derived tumor organoid growth. Organoids were quantified and their sizes were determined by H&E staining (scale bars = 100 µm, *n* = 10). H) Effects of *ATOH1* overexpression on patient‐derived GAC organoid growth. Organoids were quantified and their sizes were determined by H&E staining (scale bars = 100 µm, *n* = 10). I) Representative immunofluorescence images and quantification of *CD44* ^+^ and *SOX2* ^+^ cells among gastric epithelial cells of indicated mice at 90 days after tamoxifen administration (scale bars = 100 µm, *n* = 5). Data are represented as the mean ± SD and analyzed by Student's *t*‐test. **P<0.01, ***P <0.001 for groups connected by horizontal lines. *P*‐values < 0.05 were considered statistically significant.

Western blot and qRT‐PCR analyses revealed *ATOH1* expression in various GAC cell lines. *ATOH1* mRNA and protein levels (**Figure** **2**A; Figure S5A,B, Supporting Information) were significantly reduced in the GAC cell panel compared with those in the normal gastric epithelial GES cells. We generated AGS and NCI‐N87 cells overexpressing *ATOH1* and used lentiviral shRNA to generate SNU‐5 and Kato‐III cells with endogenous *ATOH1* knockdown (Figure S5C,D, Supporting Information).

Analysis of the differentiation trajectories of normal, *TcPP*, and *TcPP; Atoh1^fl/fl^* cohorts revealed that the absence of *ATOH1* promotes a greater proportion of cells to persist in the early stages of differentiation (Figure S6A–C, Table S5, Supporting Information). Gene scoring of *the ATOH1* ^high^ and *ATOH1* ^low^ cohorts using relative stem cell signatures from the GO biological process items in scRNA‐seq datasets from human and mouse sample revealed a negative correlation between *ATOH1* expression and pathways associated with stemness (Figure S6D,E, Supporting Information).

Gene set enrichment analysis (GSEA) of the *TcPP* and *TcPP;Atoh1^fl/fl^* cohorts revealed that the absence of *ATOH1* positively influenced the regulation of stem cell population maintenance (Figure S7A, Supporting Information). GSEA of the GEO, FJMUUH, TCGA, and AGS cohorts revealed enrichment of several genes and pathways regulating self‐renewal and stemness in patients or GAC cells with low *ATOH1* expression (Figure S7B, Supporting Information). Moreover, GSEA revealed the enrichment of signatures regulating CSCs that were also present in mouse databases (Figure S7C, Supporting Information).

We used spheroid cultures to investigate whether *ATOH1* maintains GCSC properties. Preliminary experiments revealed that these culture conditions enhanced CSC‐related properties,^[^ ^13^ ^]^ including CSC marker upregulation and enhanced tumor initiation (Figure S8A–D, Supporting Information). *ATOH1* was significantly downregulated in the spheroids compared with the parental GAC cells (Figure S8D,E, Supporting Information). *ATOH1* overexpression inhibited primary and secondary sphere formation (Figure 2B). Western blot and immunofluorescence showed that *ATOH1* overexpression downregulated the GCSC marker *CD44* and the self‐renewal marker *SOX2* in the spheroids (Figure 2C,D). In contrast, *ATOH1* knockdown significantly increased the number and size of primary and secondary spheres (Figure 2E). Western blot and immunofluorescence confirmed that *ATOH1* knockdown upregulated both *CD44* and *SOX2* in the spheroids (Figure S9A–C, Supporting Information). Flow cytometry analysis revealed that *ATOH1* expression significantly decreased the number of *CD44* ^+^ cells (Figure S9D, Supporting Information). However, *ATOH1* knockdown exhibited the opposite effect (Figure S9E, Supporting Information). A significant inverse correlation (*P* < 0.001; Figure S9F, Supporting Information) between *ATOH1* and *CD44* expression was observed in patients according to immunohistochemical (IHC) analyses.

The limiting dilution assay reduced spheroid formation capacity in *ATOH1‐*overexpressing cells from 1 in 1.14 to 1 in 3.11 (AGS cells, *P* < 0.001; Figure S9G–I, Supporting Information) and 1 in 1.23 to 1 in 3.84 (NCI‐N87 cells, *P* < 0.001; Figure S9J–L, Supporting Information). Tumor‐initiating ability is a property of CSCs.^[^ ^14^ ^]^ Serial tumor xenograft dilutions significantly lowered the tumor initiation capacity from 1 in 603611 AGS cells (control) to 1 in 3488397 cells (*ATOH1* overexpression) (*P* = 0.008; Figure 2F; Figure S10A–E, Supporting Information). Furthermore, *ATOH1*‐overexpressing NCI‐N87 cells showed lower tumorigenicity and slower tumor growth than control cells (Figure S10F–J, Supporting Information). In contrast, SNU‐5 cells with *ATOH1* knockdown showed comparatively higher tumorigenicity and faster growth rates than control cells (Figure S10K–N, Supporting Information). Therefore, *ATOH1* might regulate the GAC stemness.

Tumor‐derived organoids conserve the pathophysiology of the original tumors, while maintaining cellular heterogeneity and self‐renewal capacity.^[^ ^15^ ^]^ Organoids were established based on GAC (Figure S11A, Supporting Information) and MNU‐induced mouse tumors (Figure S11B, Supporting Information). *ATOH1* overexpression reduced the size and disrupted the architecture of organoids (Figure 2G). Similarly, *ATOH1* overexpression significantly (*P < 0.001*) compromised human organoids (Figure 2H; Figure S11C, Supporting Information).

CD44 and SOX2 are considered markers of cancer stem cell‐like properties.^[^ ^16^, ^17^ ^]^ *ATOH1* overexpression also downregulated *CD44* expression in human organoids (Figure S11D, Supporting Information). We observed significant increases in the numbers of *CD44* ^+^ and *SOX2* ^+^ epithelial cells in tumors in the *TcPP; Atoh1^fl/fl^* mouse cohort compared with the *TcPP; Atoh1^fl/+^* cohort 90 days after tamoxifen induction (*CD44* ^+^:60.5 ± 7.0% vs 43.0 ± 5.3%, *P* = 0.002; *SOX2* ^+^:82.7 ± 5.1% vs 68.6 ± 6.8%, *P* = 0.006) (Figure 2I).

### 2.4 *GAS1* is a Transcriptional Target of *ATOH1* and Contributes to *ATOH1*‐Mediated GCSC Maintenance

**Figure 3:**
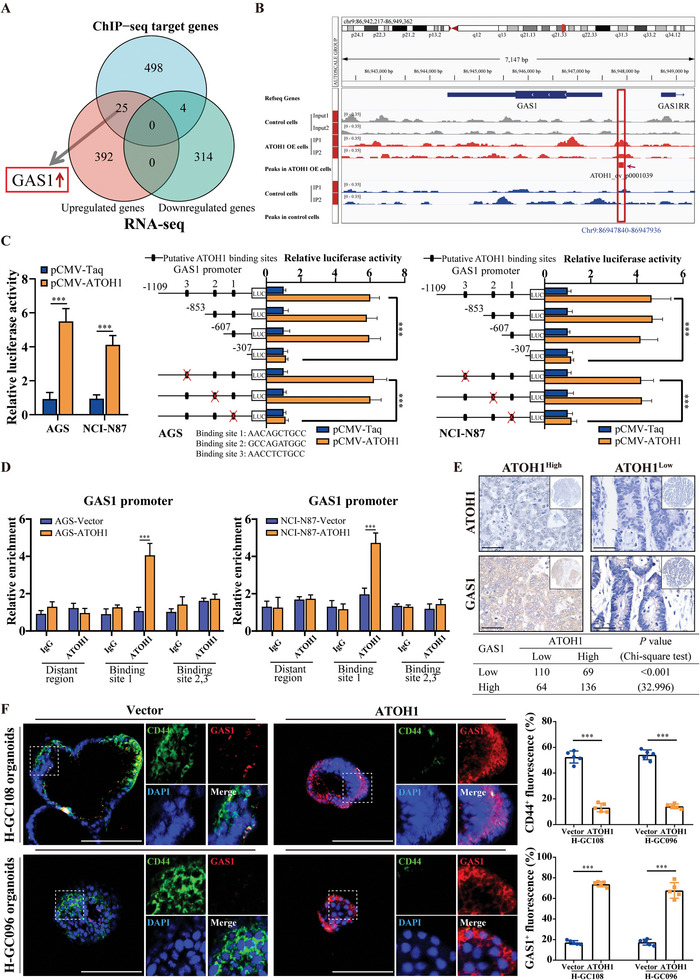
*ATOH1* upregulates *GAS1* in GAC. A) Venn diagram showing DEG overlap between RNA‐Seq and ChIP‐Seq. B) Peak signals from ChIP‐Seq indicate that *ATOH1* directly binds the *GAS1* promoter region. C) *ATOH1* transactivates the *GAS1* promoter. *GAS1* promoter construct was co‐transfected into cells via pCMV‐*ATOH1*. Relative luciferase activity was detected by luciferase reporter assay. Serial deletion and selective mutation analyses identified *ATOH1*‐responsive regions in *GAS1* promoter and relative luciferase activity were determined (*n* = 3). D) ChIP assay demonstrating direct binding of *ATOH1* to *GAS1* promoter in GAC cells (*n* = 3). E) IHC staining of *ATOH1* and *GAS1* in 379 GAC samples using tissue microarray (TMA) from FJMUUH. Correlations were analyzed by Chi‐square test (scale bars = 50 µm). F) Effects of *ATOH1* overexpression on *GAS1* and *CD44* expression in H‐GC096 and H‐GC108 patient‐derived GAC organoids. *CD44* ^+^ and *GAS1* ^+^ cells were quantified as means ± SD of five independent fields (scale bars = 100 µm). Data are represented as the mean ± SD and analyzed by Student's *t*‐test. **P <0.01, ***P <0.001 for groups connected by horizontal lines. *p*‐values < 0.05 were considered statistically significant.

ChIP‐Seq of control and *ATOH1*‐overexpressed AGS cells was used to identify genome‐wide *ATOH1*‐targeting sites, including 517 RefSeq genes (Table S6, Supporting Information). Through integrative analysis using RNA‐Seq and ChIP‐Seq data for *ATOH1*, we identified 25 upregulated genes, including *GAS1*, which bound to *ATOH1* (**Figure** **3**A). Moreover, qRT‐PCR of control and *ATOH1*‐overexpressed AGS cells showed that no compensatory molecule in addition to GAS1 was overexpressed in the growth arrest‐specific protein family (Figure S12A, Supporting Information). *GAS1* regulates cancer chemoresistance and tumorigenic potential.^[^ ^18^ ^]^ The mRNAsi‐based stemness index^[^ ^19^ ^]^ of TCGA revealed that *GAS1* expression was negatively correlated with GAC stemness (Figure S12B, Supporting Information). *GAS1* weakened the spheroid‐forming capacity of the GAC cells (Figure S12C, Supporting Information). *GAS1* overexpression downregulated *CD44* and *SOX2* expression in spheroids (Figure S12D–F, Supporting Information). The data for 48 primary tumor samples indicated that *GAS1* mRNA and protein expression levels were significantly reduced in tumor tissues (Figure S12G,H, Supporting Information). The results of the TCGA cohort was similar (Figure S12I, Supporting Information). In the GSE51105^[^ ^20^ ^]^ and GSE22377^[^ ^21^ ^]^ datasets, patients with GAC and high *GAS1* expression showed relatively better survival (Figure S12J, Supporting Information). These results suggested that *GAS1* negatively regulates GCSC.

We found that *ATOH1* mRNA expression levels were positively correlated with *GAS1* in 48 primary tumor samples (*P* < 0.001; Figure S13A, Supporting Information). Western blot and qRT‐PCR revealed that in spheroids, *ATOH1* overexpression upregulated *GAS1*, whereas *ATOH1* knockdown downregulated *GAS1* at both the mRNA and protein levels (Figure S13B,C, Supporting Information). Immunofluorescence staining showed that *ATOH1* overexpression increased the number of *GAS1* ^+^ cells (*P* < 0.001), whereas *ATOH1* knockdown had the opposite effect (*P* < 0.001) (Figure S13D, Supporting Information).

Luciferase reporter assays revealed that *ATOH1* activated the *GAS1* promoter (Figure 3C). Sequence analysis revealed three putative *ATOH1* binding sites in the *GAS1* promoter. Sequence deletion and site‐directed mutagenesis indicated that the first *ATOH1* binding site is essential for *ATOH1*‐induced *GAS1* transactivation. ChIP assay confirmed the direct *ATOH1* binding to the *GAS1* promoters in GAC cells (Figure 3D). These results suggested that *GAS1* is a direct transcriptional *ATOH1* target. IHC staining of the tissue microarray (TMA) showed that *ATOH1* was positively correlated with *GAS1* expression in human GACs (*P* < 0.001; Figure 3E). *ATOH1* overexpression upregulated *GAS1* in human organoids (Figure 3F). Rescue experiments were performed to determine whether *GAS1* contributes to *ATOH1*‐mediated GCSC maintenance. *GAS1* reduction reversed the inhibitory effect of *ATOH1* overexpression on spheroid and *CD44* ^+^ cell formation (Figures S14A,B; and S15A, Supporting Information). *GAS1* upregulation significantly reduced spheroid and *CD44* ^+^ cell formation caused by *ATOH1* knockdown (Figures S14C,D; and S15B, Supporting Information). BALB/c nude mice were subcutaneously injected with AGS cells overexpressing *ATOH1* alone, or both *ATOH1* and sh*GAS1*. *GAS1* reduction reversed the inhibitory effects of *ATOH1* overexpression on heterologous tumor growth and tumor initiation (Figure S15C–F, Supporting Information). *GAS1* upregulation reduced the tumor initiation capacity of SNU‐5‐sh*ATOH1* cells (Figure S15G, Supporting Information). These results suggested that *ATOH1* regulates GCSCs by activating the *GAS1* promoter.

### 2.5 *ATOH1* Regulates *RET*/*AKT*/*mTOR* Signaling in GAC

**Figure 4:**
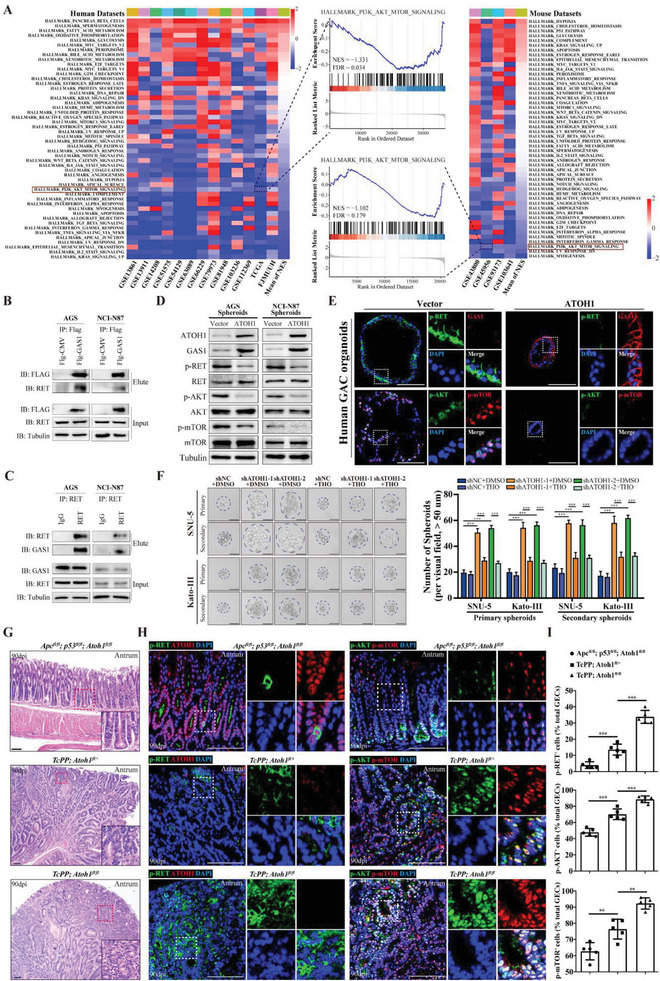
*ATOH1* regulates *RET*/*AKT*/*mTOR* signaling in human GAC cells. A) Gene set enrichment analysis (GSEA) was performed by comparing high and low *ATOH1* expression groups in TCGA, GEO, and FJMUUH human and GEO mouse GAC cohorts. Hallmark gene sets were downloaded from https://www.gsea‐msigdb.org/. B) Co‐immunoprecipitation (Co‐IP) of *GAS1* with anti‐Flag in AGS and NCI‐N87 cells identified *RET* as *GAS1* binding partner. C) Reciprocal Co‐IP confirmed protein interaction between *GAS1* and *RET* in AGS and NCI‐N87 cells. D) Western blot of *ATOH1*, *GAS1*, and *RET*/*AKT*/*mTOR* pathway members was performed on AGS and NCI‐N87 spheroids transfected with *ATOH1*. E) Representative immunofluorescence images of *GAS1*, p‐*RET*, p‐*AKT*, and p‐*mTOR* staining in patient‐derived tumor organoids (scale bars = 100 µm). F) Spheroid formation was detected in SNU‐5 and Kato‐III cells transfected with sh*ATOH1* subjected to *AKT*/*mTOR* inhibitor thioridazine hydrochloride (THO; 10 µm) (scale bars = 50 µm, *n* = 5). G) Representative H&E staining of stomachs of *Apc^fl/fl^; p53^fl/fl^; Atoh1^fl/fl^*, *TcPP; Atoh1^fl/+^*, and *TcPP; Atoh1 ^fl/fl^* mice at 90 days after tamoxifen administration (scale bars = 100 µm). H) *ATOH1*, p‐*RET*, p‐*AKT*, and p‐*mTOR* expression in stomachs of *Apc^fl/fl^; p53^fl/fl^; Atoh1^fl/fl^*, *TcPP; Atoh1 ^fl/+^*, and *TcPP; Atoh1 ^fl/fl^* mice at 90 days after tamoxifen administration (scale bars = 100 µm). I) Quantification of p‐*RET* ^+^, p‐*AKT* ^+^, and p‐*mTOR* ^+^ cells in (**H**), *n* = 5. Data are represented as the mean ± SD and analyzed by Student's *t*‐test. **P <0.01, ***P <0.001 for groups connected by horizontal lines. *p*‐values < 0.05 were considered statistically significant.

To elucidate the downstream molecular mechanism of *ATOH1* in regulating GAC, we performed GSEA on TCGA, FJMUUH, and GEO datasets. *ATOH1* cohorts with high *ATOH1* expression was compared with those with low *ATOH1* expression, and *AKT*/*mTOR* signaling was enriched in all datasets (**Figure** **4**A). Considering that *GAS1* may be a co‐receptor protein complexed with the receptor tyrosine kinase *RET*,^[^ ^22^ ^]^ we investigated whether *ATOH1* affects malignancy through *GAS1*/*RET*/*AKT*/*mTOR* signaling. We performed co‐immunoprecipitation (Co‐IP) on AGS and NCI‐N87 GAC cell lines ectopically expressing FLAG‐tagged *GAS1* and validated protein‐protein interactions. *GAS1* pull‐down assay with anti‐FLAG identified *RET* as a *GAS1* binding partner (Figure 4B). Reciprocal Co‐IP with anti‐*RET* in both cell lines revealed that *GAS1* was an interacting protein (Figure 4C). Thus, *GAS1* may combine with *RET* to form a new protein complex that inhibits *RET*/*AKT*/*mTOR* signaling. *ATOH1* overexpression significantly reduced *RET*, *AKT*, and *mTOR* phosphorylation levels in spheres (Figure 4D; Figure S16A, Supporting Information), and downregulated *RET*/*AKT*/*mTOR* phosphorylation in organoids (Figure 4E; Figure S17A–C, Supporting Information). In the xenotransplantation model, a significant inverse correlation was observed between *ATOH1* expression and *RET*/*AKT*/*mTOR* phosphorylation (Figure S17D,E, Supporting Information). In the spheres, *ATOH1* knockdown significantly augmented p‐*RET*, p‐*AKT*, and p‐*mTOR* activities (Figure S16B–D, Supporting Information). *GAS1* shRNA co‐transfection reversed the inhibitory effect of *ATOH1* overexpression on *RET*/*AKT*/*mTOR* phosphorylation. *GAS1* overexpression reversed *ATOH1* knockdown‐induced upregulation of *RET*/*AKT*/*mTOR* phosphorylation (Figure S16E–G, Supporting Information). These results suggest that *ATOH1* inhibits phosphorylation of the *RET*/AKT/*mTOR* signaling axis in a *GAS1*‐dependent manner.

To investigate whether *ATOH1* regulates GAC stemness through the *AKT*/*mTOR* signaling pathway, we added the *AKT*/*mTOR* pathway inhibitor thioridazine hydrochloride (THO) to treat developing spheroids with simultaneous *ATOH1* knockdown. THO administration significantly inhibited the increase in spheroid formation caused by *ATOH1* knockdown (Figure 4F). Western blot confirmed that THO downregulated p‐*AKT*, p‐*mTOR*, and stemness markers, which were increased by *ATOH1* knockdown (Figure S18A, Supporting Information). Thus, THO offsets the growth‐promoting effects of *ATOH1* knockdown in heterogeneous tumors in vivo (Figure S18B,C, Supporting Information).

We observed significant increases in p‐*RET* ^+^, p‐*AKT* ^+^, and p‐*mTOR* ^+^ epithelial cells in the antra of the *TcPP; Atoh1^fl/fl^* mice compared with those of the *TcPP; Atoh1^fl/+^* mice at 90 days after tamoxifen induction (p‐*RET* ^+^: 13.6 ± 3.4% vs 33.8 ± 4.0%, *P* < 0.001; p‐*AKT* ^+^: 70.1 ± 6.5% vs 88.7 ± 4.1%, *P* < 0.001; p‐*mTOR* ^+^: 76.4 ± 6.2% vs 92.3 ± 3.5%, *P* = 0.001) (Figure 4G–I; Figure S18D, Supporting Information). These results suggested that *RET*/*AKT*/*mTOR* signaling mediates *ATOH1* regulation in GAC malignancy.

### 2.6 The *ATOH1* Promoter was Hypermethylated in GAC

**Figure 5:**
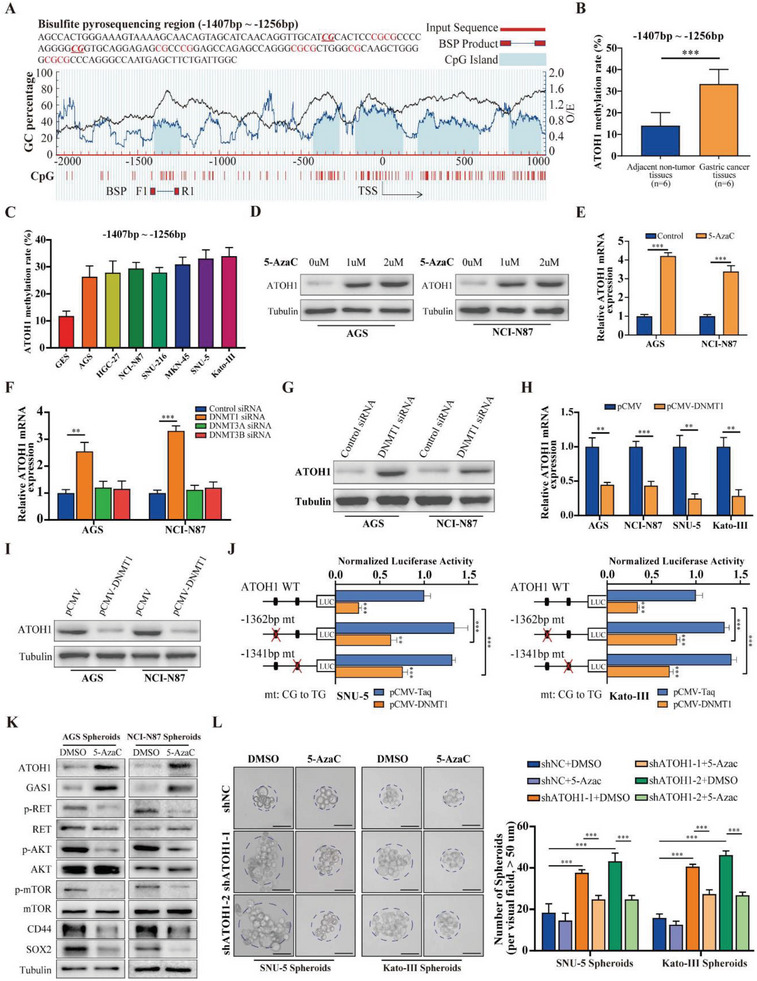
*ATOH1* promoter is hypermethylated in GAC. A) Schematic representation of CpG islands and bisulfite sequencing region in *ATOH1* promoter. Magenta font: CG sites for bisulfite sequencing; bold magenta font: most significantly altered CG site in *ATOH1*; red region: input sequence; blue region: CpG islands; black curve: trend of GAC base % content; BSP F1 and R1: bisulfite forward and reverse primer, respectively. B) Bisulfite sequencing analysis of *ATOH1* promoter region (−1,407 to −1,256 bp) and average methylation levels in adjacent non‐tumor (*n* = 6) and GAC (*n* = 6) tissues. C) Methylation levels of *ATOH1* promoter region in GES cells and GAC cell panel. D) AGS and NCI‐N87 cells were treated with 5‐AzaC at indicated concentrations for 48 h and *ATOH1* expression was measured by western blot. E) AGS and NCI‐N87 cells were treated with 1uM of 5‐AzaC for 48 h and *ATOH1* expression was measured by qRT‐PCR (*n* = 3). F) AGS and NCI‐N87 cells were transfected with DNMT siRNA for 48 h and *ATOH1* mRNA expression was measured by qRT‐PCR (*n* = 3). G) AGS and NCI‐N87 cells were transfected with *DNMT1* siRNA for 48 h and *ATOH1* expression was measured by western blot. H) GAC cells were transfected with pCMV‐*DNMT1* and *ATOH1* mRNA expression was measured by qRT‐PCR. I) AGS and NCI‐N87 cells were transfected with pCMV‐*DNMT1* and *ATOH1* expression was measured by western blot (*n* = 3). J) DNMT1 expression vector and *ATOH1* wild‐type promoter constructs or promoter constructs containing site‐specific CpG mutations were co‐transfected into SNU‐5 and Kato‐III cells. Activity levels of *ATOH1* promoter constructs containing different mutations were measured by luciferase assay. Point mutations (CG to TG) were created at CpG sites located at −1,362 and −1,341 bp (*n* = 3). K) *ATOH1*, *GAS1*, and *RET*/*AKT*/*mTOR* expression were measured by western blot in AGS and NCI‐N87 cells treated with 1 µm of 5‐AzaC. L) Spheroid formation was detected in SNU‐5 and Kato‐III cells transfected with sh*ATOH1* subjected to 1 µm of 5‐AzaC (scale bars = 50 µm). Data are represented as the mean ± SD and analyzed by Student's *t*‐test. **P <0.01, ***P <0.001 for groups connected by horizontal lines. *p*‐values < 0.05 were considered statistically significant.

Using scRNA‐seq datasets, we investigated DNA methylation levels in cancerous and normal epithelial tissues, revealing a remarkable elevation in DNA methylation in cancerous tissues compared with their normal counterparts. *ATOH1* expression could be regulated by DNA methylation. To elucidate the mechanism by which deletion of *ATOH1* regulates DNA methylation, we investigated whether *ATOH1* downregulation was related to the methylation status of its promoter in the GAC. We performed bisulfite sequencing to evaluate the *ATOH1* promoter methylation levels in six pairs of GAC and adjacent normal tissues. CpG islands and selected bisulfite sequencing regions of the *ATOH1* promoter are shown in **Figure** **5**A. The methylation levels of the CpG sites at −1,362 and −1,341 bp in the *ATOH1* promoter were significantly higher in GAC tissues than in their adjacent noncancerous tissues (Figure 5B; Figure S19A–D, Supporting Information). *ATOH1* methylation levels in GAC cell lines were significantly higher than those in normal gastric epithelial cells (Figure 5C). We treated GAC cells with the demethylation drug 5‐azacytidine (5‐AzaC) to determine whether *ATOH1* was downregulated in response to the hypermethylation of its promoter. 5‐AzaC treatment significantly increased *ATOH1* mRNA and protein levels in GAC cells (Figure 5D,E). To establish the potential roles of various DNA methyltransferases (DNMTs) in mediating *ATOH1* promoter methylation in GAC, we knocked down *DNMT1*, *DNMT3A*, and *DNMT3B* in GAC cells using specific small interfering RNAs (siRNAs) (Figure S19E, Supporting Information). Knockdown of *DNMT1* but not *DNMT3A* or *DNMT3B* rescued *ATOH1* expression (Figure 5F,G). *DNMT1* overexpression significantly inhibited *ATOH1* expression (Figure 5H,I; Figure S19F,G, Supporting Information). To determine the effects of DNA methylation on *ATOH1* promoter activity and to confirm the participation of the −1,362 and −1,341 bp CpG sites in promoter regulation, we transfected wild‐type *ATOH1* promoter constructs or those containing site‐specific CpG mutations into SNU‐5 and Kato‐III cells. *DNMT1* overexpression significantly decreased the activity of the wild‐type promoter. However, CG‐to‐TG mutations at the −1,362 and −1,341 bp CpG sites reversed the inhibitory effect of *DNMT1* on *ATOH1* promoter activity. Thus, the methylation status of the −1,362 and −1,341 bp CpG sites in the promoter region are crucial for the epigenetic regulation of *ATOH1* expression (Figure 5J). These findings suggest that *ATOH1* downregulation is associated with hypermethylation of its promoter in GAC.

We investigated whether pharmacological DNMT inhibition suppressed tumorigenesis by regulating *ATOH1*/*GAS1*/*RET*/*AKT*/*mTOR* signaling. 5‐AzaC treatment inhibited AGS tumor xenograft growth (Figure S20A–C, Supporting Information). It also upregulated *ATOH1* and *GAS1* and significantly downregulated p‐*RET*, p‐*AKT*, and p‐*mTOR* in spheroids (Figure 5K). Next, we explored whether *ATOH1* upregulation inhibits 5‐AzaC‐mediated *RET*/*AKT*/*mTOR* signaling. Kato‐III spheroids with *ATOH1* knockdown were subjected to 5‐AzaC treatment, and the effect of sh*ATOH1* on the *RET*/*AKT*/*mTOR* signaling pathway was attenuated (Figure S20D, Supporting Information). 5‐AzaC treatment weakened spheroid formation in SNU‐5 and Kato‐III cells with *ATOH1* knockdown (Figure 5L). It also significantly inhibited SNU‐5 tumor xenograft growth (Figure S20E,F, Supporting Information). These results indicate that the inhibition of *DNMT1* activity suppresses tumor growth by regulating *ATOH1*/*GAS1*/*RET*/*AKT*/*mTOR* signaling in GAC.

### 2.7 *ATOH1* Expression in Tumors is Correlated with GAC Patient Prognosis

**Figure 6:**
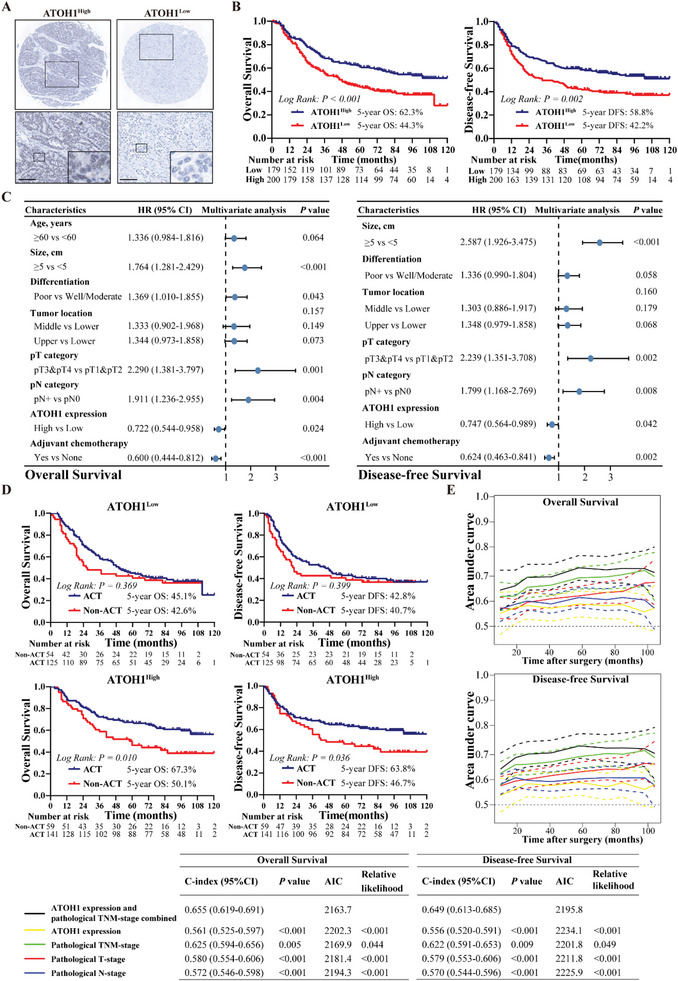
*ATOH1* expression in tumors is correlated with GAC patient prognosis. A) *ATOH1* expression in 379 paraffin‐embedded specimens of TMA from the FJMUUH cohort was determined by TMA‐based IHC staining (scale bars = 100 µm). B) Overall survival and disease‐free survival curves of GAC patients with low versus high *ATOH1* expression (*n* = 379, *ATOH1* ^Low^ = 179, *ATOH1* ^High^ = 200). C) Multivariable Cox analysis of prognostic factors for GAC patients (*n* = 379). D) Subgroup analyses of OS and DFS among GAC patients with low versus high *ATOH1* expression who received adjuvant chemotherapy or not. E) Time‐dependent receiver operating characteristic (ROC) curves comparing prognostic accuracy of *ATOH1* with pathological prognostic factors for GAC patients. Harrell's concordance index (C‐index) and Akaike information criteria (AIC) for prognostic factors were calculated and compared against those for the combination of *ATOH1* and pathological risk factors. The probability of differences in OS and DFS was ascertained by the Kaplan–Meier method with the log‐rank test.

Clinicopathological characteristics stratified by *ATOH1* expression were determined using IHC of a TMA containing 379 GAC samples from FJMUUH (Table S7, Supporting Information). Low *ATOH1* expression was significantly associated with advanced pT and pN stages. Similar results were obtained in the FHUSTC cohort (Table S8, Supporting Information). Kaplan–Meier survival analysis (**Figure** **6**A,B) revealed better five‐year overall survival (OS) in *ATOH1* ^high^ patients than *ATOH1* ^low^ patients (62.3% vs 44.3%; *P* < 0.001). *ATOH1* ^high^ patients had a significantly higher five‐year disease‐free survival (DFS) than *ATOH1* ^low^ patients (58.8% vs 42.4%; *P* = 0.002). The overall recurrence was lower in *ATOH1* ^high^ patients than *ATOH1* ^low^ patients (*P* < 0.001; Figure S21A, Supporting Information). Univariate and multivariate Cox analyses showed that *ATOH1* ^high^ status was an independent protective factor against survival (Figure 6C; Figure S21B, Supporting Information). Similar results were obtained for the OS analyses of the FHUSTC cohort and GEO datasets (Figure S21C,D, Supporting Information).

We performed a Kaplan–Meier analysis to establish whether *ATOH1* levels were associated with the prognosis of patients with GAC who had been administered adjuvant chemotherapy (ACT). Both OS and DFS were low in *ATOH1* ^low^ patients, regardless of ACT administration (Figure 6D). *ATOH1* ^high^ patients had relatively higher survival rates than *ATOH1* ^low^ patients after ACT. These findings suggest that *ATOH1* upregulation is associated with chemosensitivity and prognosis of patients with GAC. We evaluated the effect of combining *ATOH1* expression with TNM staging on the prognostic accuracy. *ATOH1* added prognostic value to clinicopathological features based on time‐dependent receiver operating characteristic (ROC), C‐index, and Akaike information criteria (AIC) analyses (Figure 6E).

### 2.8 *DNMT1*/*ATOH1*/*GAS1*/*RET*/*AKT*/*mTOR* Signaling Dysregulation Exhibits Clinical Significance

We evaluated the clinical significance of *DNMT1*/*ATOH1*/*GAS1*/*RET*/*AKT*/*mTOR* signaling in GAC. Immunohistochemistry was used to compare *DNMT1*, *ATOH1*, *GAS1*, p‐*RET*, p‐*AKT*, and p‐*mTOR* expression in a TMA comprising 92 independent primary GAC samples and adjacent normal gastric tissue. The adjacent tissues showed upregulated *DNMT1*, p‐*RET*, p‐AKT, and p‐*mTOR* and downregulated *ATOH1* and *GAS1* (Figure S22A,B, Supporting Information). Strong inverse correlations were observed between *ATOH1* and the expression of p‐*RET*, p‐AKT, p‐*mTOR*, and *CD44* (Figure S22C, Supporting Information). This finding is consistent with our in vitro and in vivo results. The combination of *DNMT1* upregulation and *ATOH1* and *GAS1* downregulation predicted shorter survival in GAC patients (Figure S22D,E, Supporting Information). These findings indicate that dysregulated *DNMT1*/*ATOH1*/*GAS1*/*RET*/*AKT*/*mTOR* signaling plays a critical role in disease progression and is a valuable prognostic biomarker for GAC.

### 2.9 THO Works Synergistically with 5‐fluorouracil (5‐FU) to Inhibit *ATOH1*‐Deficient GAC Cell Growth Both In Vitro and In Vivo

Sensitivity to 5‐FU differed significantly between *ATOH1*‐low and *ATOH1*‐high expression groups in ACRG and TCGA datasets (Figure S23A, Supporting Information). The CCK‐8 assay showed that the IC_50_ for 5‐FU treatment was significantly lower in GAC cells overexpressing *ATOH1* than in the vector cells. The IC_50_ of 5‐FU treatment was substantially higher in GAC cells with *ATOH1* knockdown than in control cells (Figure S23B, Supporting Information). *ATOH1* expression increased the 5‐FU sensitivity of the xenograft tumors (Figure S23C–E, Supporting Information). Conversely, *ATOH1* knockdown reduced xenograft tumor sensitivity to 5‐FU (Figure S23F,G, Supporting Information).

**Figure 7:**
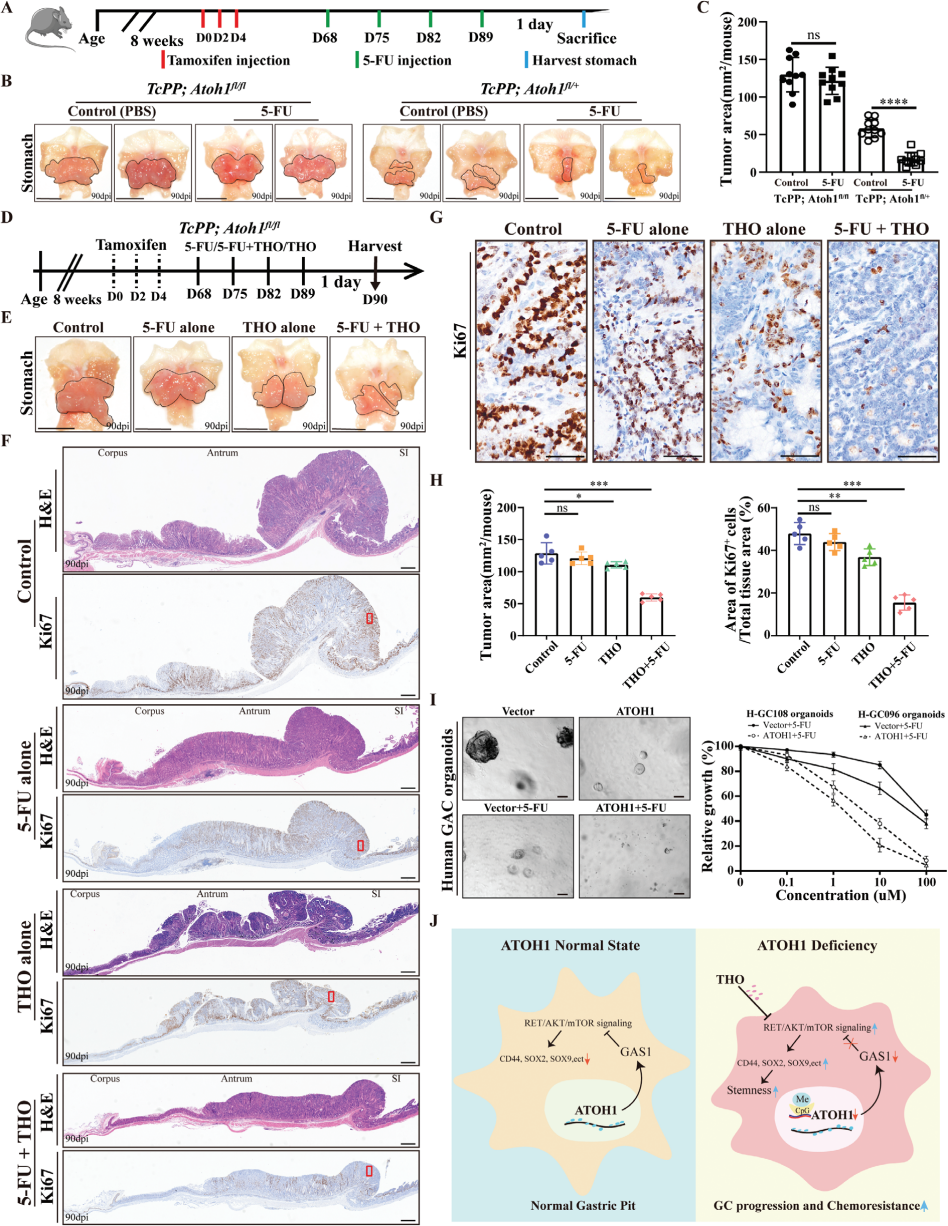
*ATOH1* controls chemoresistance in the GAC model. A) Injection timeline for tamoxifen‐induced *TcPP; Atoh1^fl/+^* and *TcPP; Atoh1^fl/fl^* mice. B) Macroscopic view of stomachs of control and 5‐FU treated mice (*n* = 10 per group) collected 90 days after tamoxifen administration. Tumors are marked by solid lines (scale bars = 1 cm). C) Total area (m^2^) of mouse tumors harvested from untreated and 5‐FU treated mice (*n* = 10 per group). D) Timelines for 5‐FU^#^, THO^##^, and 5‐FU+THO^###^ injections in tamoxifen‐induced *TcPP; Atoh1 ^fl/fl^* mice. ^#^50 mg k^−1^g BW 5‐FU weekly for 4 weeks. ^##^10 mg k^−1^g BW THO twice weekly for 4 weeks. ^###^5‐FU 50 mg k^−1^g BW 5‐FU weekly for 4 weeks plus 10 mg k^−1^g BW THO twice weekly for 4 weeks. E) Macroscopic views of stomachs of control (PBS), 5‐FU‐, THO‐, and 5‐FU+THO‐treated mice (*n* = 5 per group). Tumors are marked by solid lines (scale bars = 1 cm). F) H&E (top) and *Ki67* (bottom) expression in control, 5‐FU‐, THO‐, and 5‐FU+THO‐treated mice (scale bars = 500 µm). G) Representative images of *Ki67* expression in control, 5‐FU‐, THO‐, and 5‐FU+THO‐treated mice (scale bars = 50 µm). H) Quantification of tumor volume reduction and enumeration of proliferating cells (Ki67^+^) after 5‐FU, THO, or 5‐FU+THO treatment. I) Sensitivity of H‐GC096 and H‐GC108 patient‐derived organoids to 5‐FU after *ATOH1* overexpression (scale bars = 100 µm). J) Proposed molecular mechanism of *ATOH1* in GAC. Data are represented as the mean ± SD and analyzed by Student's *t*‐test. NS, no significance, *P <0.05, **P <0.01, ***P <0.001 for groups connected by horizontal lines. *p*‐values < 0.05 were considered statistically significant.

Sixty‐eight days after tamoxifen induction, *TcPP; Atoh1^fl/+^* and *TcPP; Atoh1^fl/fl^* mice were treated with one 5‐FU dose per week. Tissue samples from the untreated and treated mice were harvested 24 h after the final 5‐FU dose (**Figure** **7**A). In *TcPP; Atoh1^fl/+^* mice, the volumes of 5‐FU‐treated tumors were significantly lower than those of the untreated controls. However, this difference was not evident in the *TcPP; Atoh1^fl/fl^* mice (Figure 7B,C). Sixty‐eight days after tamoxifen induction, the *TcPP; Atoh1^fl/fl^* mice were treated with one 5‐FU dose per week, one THO dose twice weekly, or both 5‐FU and THO for 4 weeks. The 5‐FU+THO‐treated tumors had the smallest volumes (Figure 7D,E). There were few proliferating cells in the mice treated with THO alone and even fewer in the 5‐FU+THO‐treated mice (Figure 7F–H). The divergent efficacies of various treatment regimens for ameliorating disease progression in the *TcPP; Atoh1^fl/fl^* mouse model underscores the therapeutic value of combining *AKT*/*mTOR* inhibitors with standard chemotherapy to prevent GAC progression.

We explored the effects of altered *ATOH1* expression on the chemosensitivity of human GAC organoids. *ATOH1* overexpression rescued chemosensitivity in the organoids and inhibited their growth to a greater extent than treatment with 5‐FU alone (Figure 7I). These results demonstrated that virus‐mediated *ATOH1* overexpression inhibits in vivo tumor growth and increases GAC cell sensitivity to 5‐FU.

## 3 Discussion

Elucidating the molecular mechanisms underlying cancer stemness is essential for developing innovative strategies to overcome chemotherapy‐resistant GAC. It is also necessary to validate these strategies using preclinical models.^[^ ^23^ ^]^ Lineage tracing was used to identify all progeny stemness derived from a single cell and arrange them within the lineage hierarchy.^[^ ^24^ ^]^ In this study, we established a stomach‐specific mouse model, *Tff1‐CreERT2; Rosa26^Tdtomato^*, and empirically demonstrated by lineage tracing that *Tff1* cells seldom (if ever) exhibited stemness in gastric epithelial cells. Moreover, *Atoh1* deletion confers stemness to *Tff1* cells in a *Tff1‐CreERT2; Atoh1^fl/fl^; Rosa26^Tdtomato^* mouse model. We further elucidated the molecular mechanisms by which *ATOH1* deficiency induces CSC‐like properties that drive cancer progression in vitro and in vivo. *ATOH1* knockout inhibits *GAS1* promoter transcription and activates *RET*/*AKT*/*mTOR* signaling. The proposed molecular mechanism of action of *ATOH1* in the GAC is shown in Figure 7J. The combination of 5‐FU and the *AKT*/*mTOR* signaling inhibitor THO exhibited potential against refractory GAC, suggesting that this treatment modality might be particularly efficacious in *ATOH1*‐deficient GAC patients.

We used stomach‐specific *Atoh1* mouse models to determine the functions of *ATOH1* in the GAC and found that *Atoh1* deficiency induced CSC‐like properties and increased the tumor burden. *ATOH1*, a member of the bHLH transcription factor family, was initially considered an important regulator of cerebellar granule neuron precursors and cochlear hair cell development.^[^ ^6^, ^25^ ^]^ Subsequent studies have indicated that *ATOH1* was also implicated in cell proliferation and differentiation.^[^ ^6^, ^8^ ^]^ Prior research established intestinal‐specific *Atoh1* mouse models and showed that *ATOH1* maintained stem cell homeostasis.^[^ ^26^ ^]^ Although a recent study found that *ATOH1* could reduce the proliferation of gastric cancer cells,^[^ ^27^ ^]^ the mechanism was not explored in depth. However, the conservation of ATOH1 function in different species is unknown. We further explored the mechanism by which ATOH1 regulates CSC‐like properties in the GAC. In this study, we generated *Tff1‐CreERT2; Rosa26^Tdtomato^* mice in which gene modification occurred primarily in the pit cell lineages of the antral and corpus glands. However, *Tff1‐CreERT2* was not activated in the cecum and proximal colon. We compared *Tff1‐CreERT2; Rosa26^Tdtomato^* and *Tff1‐CreERT2; Atoh1^fl/fl^; Rosa26^Tdtomato^* mice subjected to tamoxifen induction. *Atoh1* knockout imparts stemness to *Tff1* cells. Comparison of *TcPP; Atoh1^fl/+^* and *TcPP; Atoh1^fl/fl^* mice subjected to tamoxifen induction revealed that *Atoh1* knockout increased tumor burden. *ATOH1* loss imparted stemness to gastric epithelial cells and contributed to GAC progression. Consistent results were observed in the established GAC cell lines. Chromatin immunoprecipitation sequencing revealed an *ATOH1*‐binding site in the *GAS1* promoter. *GAS1* is a multifunctional protein that induces apoptosis and regulates cell‐cycle arrest in various tissues.^[^ ^28^ ^]^ Here, *GAS1* is identified as a novel *ATOH1* target gene that regulates cancer stemness. *ATOH1* may upregulate *GAS1* by activating *GAS1* promoter transcription. Earlier studies have reported that *GAS1* expression suppresses tumor progression by inhibiting cell proliferation in GAC.^[^ ^18^ ^]^ Recent evidence has indicated that *GAS1* regulates CSCs.^[^ ^29^ ^]^ *GAS1* is structurally homologous with glial cell line‐derived neurotrophic factor (GFRαs) receptors^[^ ^30^ ^]^ and complexes with *RET*. This complex promotes cell survival and proliferation by activating the *MAPK* and *PI3K*/*AKT* signaling pathways.^[^ ^31^ ^]^ In GAC cells, *GAS1* prevents Tyr1062 phosphorylation of *RET* by complexation. GSEA revealed that *ATOH1* might regulate GAC stemness through *AKT*/*mTOR* signaling. *PI3K*/*AKT*/*mTOR* signaling may be critical in various solid tumors as it regulates tumor cell growth, chemoresistance, metabolism, and CSC.^[^ ^32^ ^]^ This study indicates that *GAS1* is vital as an *ATOH1* transcription target and reduces GCSC activity and chemoresistance via the *RET*/*AKT*/*mTOR* signaling axis. Our preclinical model revealed that the combination of chemotherapy with drugs targeting *AKT*/*mTOR* signaling overcame *ATOH1* deficiency‐induced chemoresistance. The combination of 5‐FU with drugs targeting CSCs may be a promising strategy to overcome chemotherapy resistance in patients with GAC. Clinically validating the safety and efficacy of molecular markers targeting *ATOH1* deficiency in treating GAC and routinely utilizing this approach in routine GAC therapy are needed.

*ATOH1* deficiency remains a problem in GAC progression. Nonetheless, we demonstrated that it modulates the expression of genes and pathways that regulate cellular transformation and cancer progression. Epigenetic programs regulate gene expression and CSC self‐renewal and differentiation.^[^ ^33^ ^]^ Abnormal DNA methylation is a common epigenetic regulatory defect in various tumors.^[^ ^34^ ^]^ DNA hypermethylation in CpG islands may cause a loss of differentiation in state‐specific gene expression and the rescue of stemness. CpG methylation is catalyzed by DNMTs including *DNMT1*, *DNMT3a*, and *DNMT3b*.^[^ ^35^ ^]^ Previous studies have shown that DNMTs maintain stem cells, progenitor cells, and CSCs.^[^ ^34^, ^36^ ^]^ However, the molecular mechanisms through which DNMTs regulate GCSCs remain unknown. Bisulfite sequencing analysis of GAC tissues revealed that *DNMT1* downregulated *ATOH1* and significantly increased the methylation levels of CpG sites at −1,362 and −1,341 bp in the *ATOH1* promoter. *DNMT1* is a methylation‐maintenance enzyme that regulates the genomic integrity and transcription of certain genes and retrotransposons.^[^ ^37^ ^]^ We showed that *DNMT1* prevented *ATOH1* upregulation and suppresses the properties of CSC‐like cells.

## 4 Conclusion

In summary, we determined that *DNMT1*‐mediated hypermethylation leads to *ATOH1* deficiency by blocking *GAS1* promoter transcription. This, in turn, activates *RET*/*AKT*/*mTOR* signaling to acquire CSC‐like and chemoresistant properties in GAC cells, resulting in poor GAC prognosis.

## 5 Experimental Section

### Animal Studies

All animal experiments were performed in accordance with the protocols approved by the Animal Experimentation Ethics Committee of Fujian Medical University (IACUC FJMU 2021‐0280).

*Mice*: *Rosa26‐LSL‐Tdtomato* (Cat# 007914), *Apc^fl/fl^* (Cat# 029275), and *p53^fl/fl^* (Cat# 008462) mice were purchased from Jackson Laboratories (Bar Harbor, ME, USA). *Tff1‐CreERT2*, *Atoh1^fl/f^* ^l^, and C57BL/6 wild‐type mice were purchased from Cyagen Biosciences Inc. (Santa Clara, CA, USA). Mouse gene sequences are listed in Table S1 (Supporting Information). Mice were housed under specific pathogen‐free conditions. Age‐ and sex‐matched littermates ≥ 6–8 weeks old were used in the experiments. Mice were intraperitoneally injected with tamoxifen (T832955; MACKLIN, Shanghai, China) dissolved in sunflower oil at the time points indicated in the text and/or figures. Samples were analyzed at the time points indicated in the text and/or figures. Additional materials and methods are described in Supplementary Information.

*N‐Nitroso‐N‐methylurea (MNU)‐Induced Mice*: A mouse model of MNU‐induced GAC (HY‐34758; MCE, Monmouth Junction, NJ, USA) was established as previously described with slight modifications.^[^ ^38^ ^]^ Briefly, mice were given drinking water containing 240 ppm MNU on alternate weeks for a total of 5 weeks (total exposure of 3 weeks).

### Organoid Culture

Organoid cultures of human and mouse GAC were prepared according to a previously published protocol.^[^ ^39^ ^]^ Briefly, tumor tissues from the stomach were washed twice with PBS containing 1 × penicillin/streptomycin (BL505A, Biosharp, Hefei, China), followed by the removal of the muscle layer and mucus using scissors, and cut into 2–3 mm pieces followed by digestion with 2.5 mg ml^−1^ Collagenase A (Sigma Aldrich, St. Louis, MO, USA) for 30 min. Five milliliters of dissociation buffer, including d‐sorbitol (Sigma Aldrich) and sucrose (Sigma Aldrich), were added to the tissue and shaken for 2 min. The final supernatant was passed through a 70 µm filter, and the crypt fraction was centrifuged at 150 g for 5 min. After washing with ice‐cold PBS, the gland pellet was resuspended in Matrigel (356255, Corning, Corning, NY, USA) supplemented with standard gastric organoids [advanced DMEM/F12 (#12634010, Thermo Fisher Scientific, Waltham, MA, USA), 1× GlutaMax (#35050061, Thermo Fisher Scientific), 1× HEPES (#15630080, Thermo Fisher Scientific), 1× Penicillin/Streptomycin, 50% Wnt3a, 10% RSPO‐1, 10% Noggin, 1× B27 (#17504001, Thermo Fisher Scientific), 50 ng mL^−1^ EGF (PHG0311, Thermo Fisher Scientific), 200 ng mL^−1^ FGF10 (#100‐26, Peprotech, Rocky Hill, NJ, USA), 1 mm N‐acetyl‐L‐cysteine (#A9165, Sigma Aldrich), 1 nm Gastrin (#G9145, Sigma Aldrich), 2 mm A83‐01 (#2939/10, Tocris, Bristol, UK), 10 mm Y‐27632 (#1254/10, Tocris)]. Finally, 50 µl Matrigel suspension was carefully ejected into the center of each well of a 24‐well plate. Standard gastric organoid medium (1 mL) was added to each well. The organoids were cultured in a 5% CO_2_ incubator at 37 °C and changed media every 2–3 days. Organoids from the second passage were infected with lentivirus with control or ATOH1 overexpression in 15 ml tubes overnight. The diameter and number of organoids in three random 100× magnification fields were measured under a light microscope 7 days after infection. For histological examination, the organoids were fixed in 4% paraformaldehyde for 1 h, embedded in 2% agarose gel, or directly fixed in Matrigel in formalin for the generation of paraffin blocks, sectioning, and staining.

### Statistical Analysis

Statistical analyses were performed using SPSS software (version 22.0; IBM Corporation, Armonk, NY, USA), GraphPad Prism version 8.0 (GraphPad Software, La Jolla, CA, USA), and R software environment, version 4.2.1 (R Foundation for Statistical Computing, Vienna, Austria). Continuous variables are expressed as mean (standard deviation), and categorical variables are expressed as numbers. Differences between groups were assessed using the *t*‐test or χ^2^ test, as appropriate.

Overall survival (OS) was defined as the time from surgery to death from any cause. Disease‐free survival (DFS) was defined as the time from surgery to recurrence or death from any cause. Survival curves were estimated using the Kaplan–Meier method, and the log‐rank test was used to determine statistical significance. Prognostic factors were examined using univariate and multivariate analyses with the Cox proportional hazards model. Harrell's concordance index (C‐index) was used to measure the discriminatory ability of different prognostic models.^[^ ^40^ ^]^ The Akaike information criterion (AIC) within the Cox regression model was used to compare the performances of different prognostic models; smaller AIC values represented a better optimistic prognostic stratification.^[^ ^41^ ^]^ The relative likelihood of the two models was calculated using the following formula: exp ((AIC (model A)–AIC (model B))/2). The relative likelihood represents the probability that model A minimizes information as effectively as model B and can thus be interpreted as a *p*‐value for the comparison of both AIC values.^[^ ^42^ ^]^ A time‐dependent receiver operating characteristic (ROC) analysis was also performed to assess the discriminatory power of the prognosis model for time‐dependent disease outcomes.^[^ ^43^ ^]^

Images from all representative histological experiments, western blot, and IF were obtained at least three times independently. All tests were 2‐sided with a significance level of *P* < 0.05. *P < 0.05; **P < 0.01; ***P < 0.001, ****P < 0.0001.

### Ethical Statement

Human tissue samples: All de‐identified gastric adenocarcinoma (GAC) tissues were obtained from the Fujian Medical University Union Hospital (FJMUUH, Fuzhou, China), the First Affiliated Hospital of University of Science and Technology of China (FHUSTC, Hefei, China), and the Qinghai Provincial People's Hospital (QHPH, Xining, China). The institutional review committee has approved all experimental protocols using de‐identified human specimens of each institution (No. 2022KY035, No. 2020‐WCK‐01, and No. 2020‐42). Informed consent was obtained from subjects in this experiment. The study complied with the principles outlined in the Declaration of Helsinki. We constructed 3 tissue microarrays (TMA) of 379 cases of tumor tissues and 3 TMA of adjacent non‐tumor gastric tissues (more than 5 cm away from tumor margin) from FJMUUH between 2010 and 2015. A total of 182 paraffin‐embedded samples of GAC tissues were obtained at the FHUSTC between 2013 and 2014 and were used for validation of clinical prognostic and correlation analysis. The inclusion criteria were as follows: (a) histological identification of GAC; (b) the absence of combined malignancy and distant metastasis; (c) availability of complete follow‐up data. All the cases were restaged according to the criteria described in the AJCC cancer staging manual (8th edition). We also collected GAC tumor tissues and adjacent non‐tumor gastric tissues from 14 GAC patients from the FJMUUH, 8 patients from the FHUSTC, and 5 patients from the QHPH with complete clinicopathological features for transcriptomic RNA sequencing. Furthermore, fresh GAC samples were collected from 16 patients who had received chemotherapy at FJMUUH for RNA sequencing, 8 of whom were chemosensitive and 8 were chemoresistant. In this study, progressive disease or stable disease after 4 cycles of chemotherapy stipulated by the revised RECIST guideline was defined as chemoresistant; complete response or partial response after 2 cycles of chemotherapy stipulated by the revised RECIST guideline was defined as chemosensitive. According to the GAC treatment guidelines, a 5‐Fluorouracil (5‐FU) based chemotherapy regimen was recommended for the 16 patients.

## Conflict of Interest

The authors declare no conflict of interest.

## Author Contributions

Q.Z., H.‐G.W., J.‐H.Y., and R.‐H.T. contributed equally to this work and should be considered co‐first authors C.‐M.H. had full access to all data in the study and takes responsibility for the integrity of the data and the accuracy of the data analysis. Q.C., Q.Z., H.W., C.H., and C.Z. contributed to concept and design. Q.Z., Q.C., H.W., J.Y., A.L., Z.L., X.H., Y.L., H.Z., G.L., Z.H., K.X., W.Q., Y.Z., M.J., Q.H., Z.S.‐G., P.L., and J.X performed acquisition, analysis, and interpretation of data. Q.Z., H.W., Q.C., J.Y., R.T., and C.H. drafted the manuscript. Q.Z., H.W., and J.Y. performed statistical analysis. Q.Z., Q.C., H.W., J.Y., G.Z., Q.Z., A.L., Z.L., X.H., Y.L., G.L., Z.H., K.X., W.Q., Y.Z., J.L., R.T., Z.H., J.H., P.L., and J.X. provided administrative, technical, or material support. Q.Z., R.T., and Q.C. performed supervision.

## Supporting information

Supporting Information

## Data Availability

The data that support the findings of this study are available from the corresponding author upon reasonable request.
