# Supplementary material for: Loss of ATOH1 in Pit Cell Drives Stemness and Progression of Gastric Adenocarcinoma by Activating AKT/mTOR Signaling through GAS1
Source: Adv Sci (Weinh). 2023 Oct 12;10(32):2301977. doi: 10.1002/advs.202301977 (PMC10646280; doi:10.1002/advs.202301977)
Supplement: Supplementary file 1 — Supporting Information [file ADVS-10-2301977-s001.pdf]

ADVANCED SCIENCE
Open Access

Supporting Information

for *Adv. Sci.*, DOI 10.1002/advs.202301977

Loss of ATOH1 in Pit Cell Drives Stemness and Progression of Gastric Adenocarcinoma by Activating AKT/mTOR Signaling through GAS1

Qing Zhong, Hua-Gen Wang, Ji-Hong Yang, Ru-Hong Tu, An-Yao Li, Gui-Rong Zeng, Qiao-Ling Zheng, Zhi- Yu Liu, Zhi-Xin Shang-Guan, Xiao- Bo Huang, Qiang Huang, Yi-Fan Li, Hua-Long Zheng, Guang-Tan Lin, Ze-Ning Huang, Kai-Xiang Xu, Wen-Wu Qiu, Mei-Chen Jiang, Ya-Jun Zhao, Jian-Xian Lin, Zhi-Hong Huang, Jing-Min Huang, Ping Li, Jian-Wei Xie, Chao-Hui Zheng, Qi-Yue Chen* and Chang-Ming Huang**

Materials and Methods

Follow-up

All patients were recommended standard postoperative follow-up,[1] including visits every 3-6 months for the first 2 years, every 6-12 months from the 3rd to 5th year, and annually thereafter. Most routine follow-up appointments included a physical examination, laboratory testing, chest radiography, abdominopelvic ultrasonography or computed tomography, positron emission tomography-computed tomography (PET-CT) as appropriate, and an annual endoscopic examination was also performed. Recurrence was identified by medical history and physical examination in combination with imaging evaluation, cytology, or tissue biopsy (preferred when feasible). Recurrences were categorized as locoregional, peritoneal, or distant.[2]

Tissue microarray (TMA)

A series of TMAs containing GAC samples were constructed.[3] Briefly, all GAC tissues were reviewed by pathologists, and representative areas free from necrotic and hemorrhagic materials were marked in paraffin blocks. For each sample, a 1.5-mm core was punched from the donor blocks and transferred to the recipient paraffin block at defined array positions using a TMA instrument. Several serial sections (4μm in thickness) were cut from all TMAs, and one section was stained with H&E (hematoxylin and eosin) as a reference.

Lentiviral particles, and plasmid transfection

Overexpression and shRNA lentiviruses for *ATOH1* and *GAS1* as well as control lentivirus were purchased from GeneChem Corporation (Shanghai, China) and Applied Biological Materials Inc. (Richmond, Canada). Co-transfection was performed according to the manufacturer's instructions. Puromycin (2 μg/ml, Sigma-Aldrich, St. Louis, MO, USA) was used to select stable clones for at least 2 weeks. At the indicated time points, the cells were harvested for mRNA and protein analysis as well as for other assays. *DNMT1, DNMT3a,* and *DNMT3b* siRNA plasmids as well as control siRNA plasmids were purchased from Santa Cruz Biotechnology (Heidelberg, Germany). The expression plasmid for pCMV-*DMNT1* and pCMV empty plasmid was obtained from OBIO Technology Co., Ltd. (Shanghai, China). For transient transfections, Polyjet and LipoJet reagents were used for DNA and siRNA transfection, respectively, following the manufacturer's instruction (Signagen Laboratories, Frederick, MD, USA). The transient transfection of the indicated doses of siRNA or plasmids was performed in 6-well plates. The cells were collected for 48h for plasmids or 72h for siRNA after transfection. The infection efficiency was validated using qRT-PCR or western blotting assays.

Transcriptomic RNA sequencing

Total RNA was extracted with TRIzol™ reagent (#15596018, Invitrogen, Carlsbad, CA, USA). Transcriptomic RNA-Seq was performed at KangChen Biotech Inc. (Shanghai, China). Briefly, a total of 1 ~ 2μg of RNA per sample was used to construct a cDNA library. The quality of the constructed library was evaluated using an Agilent 2100 Bioanalyzer (Agilent Technologies, Santa Clara, CA, USA). Library sequencing was performed on the Illumina HiSeq 4000 platform (Illumina, San Diego, CA, USA).

Analysis of single-cell RNA sequencing (scRNA-seq) data

Local samples were obtained from the Department of Gastric Surgery in FJMUUH. The cancer tissue samples were derived from eight GAC patients with primary gastric tumors that underwent GAC resection. The adjacent paracarcinoma gastric tissues were taken more than 5 cm away from the cancerous tissues. Mouse gastric cancer tissue samples were collected from *TcPP* and *TcPP;Atoh1$^{fl/fl}$*, following a 90-day period of tamoxifen induction. Droplet-based 3' single-cell RNA-Seq (10x Genomics) was performed at the SMF core. Transcripts were mapped and assigned to individual cells by barcodes using Cell Ranger. FASTQ files from published data were downloaded from NCBI (GSE183904) and (GSE216139).[4] Cellranger v3.1.0 software (10x Genomics) was used to process FASTQ files and obtain Unique molecular identifier (UMI) counts with the GRCh38/hg38 genome as a reference. Further analysis was performed using the Seurat package in R (v4.2.1). Low-quality cells were filtered by determining UMI counts, the numbers of transcripts, and the percentage of mitochondrial transcripts. NormalizeData and FindVariableFeatures functions were used for UMI counts normalization and variable features. Integration anchors were identified by the FindIntegrationAnchors command to integrate the two groups (gastric carcinoma and paracarcinoma), and data were integrated by the IntegrateData command. ScaleData function was applied before dimensional reduction analysis. Principal component analysis (PCA) was performed, and non-linear dimensional reduction t-Distributed Stochastic Neighbor Embedding (t-SNE) was applied to visualize the data. Cell clusters were identified by FindNeighbors and FindClusters functions. Epithelial cell subset was selected for re-clustering and specific cell types were identified based on the markers as previously described.[5] Cell-cell interaction (CCI) analysis was performed by python package CellPhoneDB v4, while pseudotime trajectories were inferred by Monocle 2.

Gene set enrichment analysis

To analyze the *ATOH1*-associated signaling pathways, the patients were divided into three groups (low, median, and high) by the value of *ATOH1* expression in a tertile way, the mice were divided into two groups (high and low) by mean of *ATOH1* expression, the cells were divided into the two groups (vector and *ATOH1*) according to virus transfect. The differential expressed genes between the two groups were identified by using the "limma" package. Normalization Enrichment Score (NES) and the false discovery rate (FDR) of each gene set were calculated using the "clusterProfiler" package.[6] The reference gene sets, including hallmark gene sets/curated gene sets and GO biological processes gene sets were downloaded from Gene Set Enrichment Analysis (GSEA) (https://www.gsea-msigdb.org/). Each gene set was considered significant when the FDR was less than 25%.

mRNAsi-based stemness index

The mRNA expression-based signature contains a gene expression profile comprising 11,774 genes,[7] the workflow to generate the stemness indices (mRNAsi) is available on https://bioinformaticsfmrp.github.io/PanCanStem_Web/.

Drug sensitivity

We calculated the sensitivity of each GAC patient to 5-FU through the 'oncoprdict' R package[8] based on the gene expression data (log2(FPKM + 1)) from the TCGA pan-cancer dataset (https://portal.gdc.cancer.gov/) and the ACRG (GSE62254) dataset[9] (https://www.ncbi.nlm.nih.gov/gds/).

Chemoresistance Assay

Chemotherapy-induced cytotoxicity was determined by CCK-8 cell proliferation assay (CT01C, Cellcook, Guangzhou, Guangdong, China). Briefly, 1×10^4 cells/well were seeded in 96-well plates, allowed to attach overnight, and then chemotherapeutic agents 5-FU (HY-90006, MCE, Monmouth Junction, NJ, USA) were added to the complete culture medium, at various concentrations. CCK-8 assay was performed according to the manufacturer's instructions. The relative number of viable cells as compared to the number of cells without drug treatment was expressed as percent cell viability. All results in the study were based on at least three parallel measurements each time and each measurement were repeated in up to two independent experiments.

Tumor spheroid culture

Cells were seeded into ultra-low attachment 6-well dishes (Corning Life Sciences, NY, USA) and cultured in serum-free DMEM-F12 containing 20 ng/ml epidermal growth factor (EGF), 10 ng/ml basic fibroblast growth factor (bFGF), 2% B-27 (Life Technologies, Gaithersburg, MD, USA), and 2 mM L-glutamine (Life Technologies, Gaithersburg, MD, USA) as previously described.[10] Spheroids were incubated in a 5% CO_2 chamber at 37°C for seven days. The culture medium was changed every three days. The diameter and number of tumor spheres in three random magnification fields were calculated under All-in-one Fluorescence Microscope (BZ-X700, Keyence Corp, Atlanta, GA, USA) in the bright light model. Spheroids were collected after 7 days except when noted otherwise. Protein was extracted for analysis, or cells were dissociated with Accutase (Innovative Cell Technologies, San Diego, CA, USA) and used for other experiments.

In vitro limiting dilution assay

In vitro limiting dilution assay was performed using U-bottom 96-well plate as described previously.[10] AGS and NCI-N87 *ATOH1*-overexpression or control cells were diluted into three groups with different cell concentrations (1, 10, 100 cells/well). After 3 weeks of incubation, the number of wells with spheres was calculated. The frequency of spheroid formation was calculated using the Extreme Limiting Dilution Analysis (http://bioinf.wehi.edu.au/software/elda/) at 21 days post-incubation.

Quantitative real-time PCR (qRT-PCR)

Total RNA was extracted using Trizol (Invitrogen, Carlsbad, CA, USA) according to the manufacturer's instructions. Subsequently, an Advantage RT-for-PCR kit (Clontech Laboratories Inc, Mountain View, CA, USA) was used for cDNA synthesis. The public database PrimerBank (https://pga.mgh.harvard.edu/primerbank/) was used to obtain gene-specific primers (Table S2), each pair of primers spanning 2 different exons and 1 intron. ICycler (Bio-Rad Laboratories, Hercules, CA, USA) was used to perform qPCR to measure mRNA levels. The data were analyzed using the $2^{-\Delta\Delta CT}$ method with HPRT1 (human) as a control.

Dual-luciferase reporter assay

The luciferase reporter assay was performed with the Dual-Luciferase Reporter Assay System (E1960, Promega, Madison, WI, USA) according to the manufacturer's protocol. Relative luciferase activity was measured using a Microplate Reader (Spark 10M, TECAN, Switzerland) and the Firefly luciferase activity was normalized to Renilla luciferase activity to reflect transfection efficiency. Experiments were performed in triplicate.

Co-immunoprecipitation (Co-IP)

Co-immunoprecipitation (Co-IP) assay was used to detect the interaction between proteins. Cells stably expressing *GAS1*-FLAG or empty vector were lysed in ice-cold lysis buffer (50 mM Tris-Cl (pH 7.4), 150 mM NaCl, 0.5% Triton X-100, 5 mM EDTA, 1× Protease and Phosphatase inhibitors). The total proteins (1–5 mg) were immunoprecipitated using 2 µg of anti-Flag (14793, CST, Danvers, MA, USA), anti-Ret (ab134100, Abcam, Cambridge, UK) antibodies, together with Protein A/G Magnetic Beads (Merck Millipore, Billerica, MA, USA). After washing with washing buffer six times, immunoprecipitated proteins were eluted by heating in loading buffer at 100 °C for 10 mins and then subjected to SDS-PAGE for Western blotting.

Immunofluorescence (IF)

IF staining were performed as described previously[10]. Briefly, paraffin-embedded tissues were deparaffinized in Histo-Clear (National Diagnostics, Atlanta, GA, USA) and Histo-Clear was removed in gradient ethanol. Next, the citric acid buffer was used for antigen retrieval. 5% donkey serum (SL050, Solarbio, Beijing, China) and 0.2% Triton X-100 (ST795, Beyotime, Shanghai, China) were used as blocking reagents. They were fixed with 4% paraformaldehyde for cell sides, permeabilized, and blocked with 5% donkey serum and 0.2% Triton X-100. The primary antibody (Table S3) was incubated overnight at 4 °C. Some primary antibodies were listed as follows: Anti-ATOH1 (PA5-29392, Invitrogen, Carlsbad, CA, USA); Anti-*GAS1* (DF4098, Affinity Biosciences, Beijing, China); Anti-Phospho-mTOR (Ser2448) (67778-1-Ig, Proteintech, Wuhan, Hubei, China); Anti-Phospho-Akt (Ser473) (ET1607-73, HUABIO, Hangzhou, Zhejiang, China); Anti-*SOX2* (ab171380, Abcam, Cambridge, UK); Anti-*CD44* (ab157107, Abcam, Cambridge, UK). After washing 3 times with 1× PBS (sections) or 1× PBS containing 0.1% Triton X-100 (PBST, whole-mount samples), slides were incubated with 1: 200 Alexa Fluor-488 or Alexa Fluor-555 or Alexa Fluor-647 (Alexa Fluor-conjugated; Abcam, Cambridge, UK) secondary antibody for 2 hours in the dark. Finally, the nuclei were stained with DAPI (Vector Laboratories, Burlingame, CA, USA). The Leica SP5 microscope (Leica, Hesse-Darmstadt, Germany) was used to acquire images. Immunoreactivity was evaluated by two investigators and discordant cases were subsequently discussed and agreed upon.

Immunohistochemistry (IHC)

According to PV-9000 General Two-step Detection Kit (Zhong Shan-Golden Bridge Biological Technology Co., Ltd., Beijing, China) instructions constructed IHC staining. Sections were then counterstained with hematoxylin, dehydrated, and mounted. Staining intensities and extents of *ATOH1*, *GAS1*, p-*RET*, p-*AKT*, p-*mTOR*, and *CD44* expression were graded as follows: negative (score 0), weak (score 1),

moderate (score 2), and strong (score 3). Percentage scores were assigned as 1, 1–25%; 2, 26–50%; 3, 51–75%; and 4, 76–100%. The scores of each tumor sample were multiplied to give a final score of 0–12, and the tumors were finally determined as lower expression, score ≤ 4; and high expression, score > 4. Immunohistochemical scores were evaluated by two independent pathologists, and in case of disagreement, a third pathologist would be invited to discuss and reach a unanimous decision.

Flow Cytometry

For detection of the *CD44* proportion, 70%-90% confluent cells in a 100-mm cell plate (5-10 million cells per plate) were washed once with 1×PBS, and then cells were dissociated from plates using Trypsin-EDTA (Invitrogen, Carlsbad, CA, USA) or nonenzymatic solution Cell stripper (Mediatech Inc., Manassas, VA, USA) and centrifuged. Cell pellets were resuspended and incubated for 30 min at room temperature with a 1000-fold dilution of the following antibodies: Allophycocyanin (APC)-conjugated *CD44* (17-0441-82, Thermo Fisher Scientific, Waltham, MA, USA) mouse monoclonal (clone G44-26; BD Biosciences, San Jose, CA, USA). Then samples were washed by 1×PBS 3 times and resuspended in 500 μl of 1×PBS and analyzed using BD LSRFortessaX-20 flow cytometer (Becton, Dickinson and Company, Franklin Lakes, NJ, USA). APC Rat IgG2b κ Isotype Control (17-4031-82, Thermo Fisher Scientific, Waltham, MA, USA) was used as a control. The results were analyzed using the software FlowJo version 7.2.4 (Tree Star Inc., Ashland, OR; http://www.treestar.com).

Chromatin immunoprecipitation (ChIP) assays

The ChIP and ChIP-Seq analysis were performed by Igenebook Bioinformatics Institute (Wuhan, Hubei, China). Briefly, 1×10^7 cells were prepared and crosslinked with 1% formaldehyde (Sigma-Aldrich, St. Louis, MO, USA) for chromatin extraction. After sonication, fragmented DNA was obtained and then incubated with antibody (anti-*ATOH1*, 21215-1-AP, Proteintech, Wuhan, Hubei, China)-coated beads for 12 h at 4°C. After extensive washing, 20 ml 5M NaCl was added into immunoprecipitated chromatin to de-cross-linked overnight, and the concentration of purified de-cross-linked product was measured for subsequent sequencing. Enriched DNA fragments were subjected to library preparation and sequencing was constructed on Illumina HiSeq 2000 with PE 150 method. Clean data were filtrated using FastQC (version: 0.11.5) and mapped to the reference genome (https://www.ncbi.nlm.nih.gov/genome/) by BWA (version: 0.7.15-r1140). "ChIPseeker" R package was applied to draw a vennpie diagram of the distribution of Reads on gene functional elements. Deeptools (version: 2.5.4) was used to describe the read density distribution. MACS was used to analyze peak information in the genome, and the threshold for screening the significant Peak was a value of q <0.05. Peaks with FDR value <0.05 and Fold > or <0 in both replications were considered as differentially expressed peaks, and Integrated Genome Viewer was utilized to generate signal plotting of individual genes. For ChIP analysis, qRT-PCR was used to analyze the binding of *ATOH1* to the promoter of a *GAS1* target gene.

Western blot

The total protein of cells was lysed with RIPA buffer (Beyotime, Shanghai, China) containing Protease and Phosphatase inhibitors. The concentration of protein was quantified using the Pierce™ BCA Protein Assay Kit (Thermo Fisher Scientific, Waltham, MA, USA). 30 μg of protein samples were separated by 10% SDS-PAGE and transferred to a PVDF membrane (Millipore, Billerica, MA, USA). The membrane was blocked in 5% non-fat milk for one hour at room temperature and incubated with diluted primary antibody at 4°C overnight. Next, the membrane was incubated with horseradish peroxidase (HRP)-labeled secondary antibody for one hour at room temperature. The signal was visualized using ECL reagent and photographed by ChemiDoc Touch Imaging System (BioRad, Hercules, CA, USA). The details of primary antibodies are listed in Table S3.

Bisulfite sequencing analysis

Genomic DNA from FFPE, fresh-frozen tissues, and cells were isolated using a Wizard DNA Clean-Up system, according to the manufacturer's instructions. A BisulFlash DNA Modification Kit (BersinBio Co., Ltd., Guangzhou, Guangdong, China) was applied to conduct the bisulfite modification of DNA (1-2 mg). Meth Primer 2.0 (http://www.urogene.org/methprimer2) was used to design the bisulfite sequencing primers. The primer sequences are shown in Table S2. The BiQ Analyzer v2.0 was utilized for the sequencing reaction and methylation level quantification.

***In vivo* xenograft mice model**

All BALB/c nude mice (4-6 weeks of age) used in our study were purchased from Beijing Vital River Laboratory Animal Technology Co., Ltd.

To evaluate the effect of *ATOH1* on stemness, limiting dilution assays were performed in nude mice. Cells were injected subcutaneously into the right axillary fossa of nude mice at indicated cell concentrations. Five mice were used in each experimental group. Tumor formation was checked every 3-4 days and the mice were sacrificed at 4-6 weeks after injection and the tumors were weighed and used in immunohistochemical staining studies. Tumor volume was calculated with the following formula: $V = (L \times W^2)/2$ (V, tumor volume; L, length; W, width), and growth curves were plotted using average tumor volume within each experimental group at the set time points. The frequency of tumor-initiating cells was calculated using the extreme limiting dilution analysis program (http://bioinf.wehi.edu.au/software/elda/).

To evaluate the effect of thioridazine hydrochloride (THO) on tumor growth, 2×10^6 SNU-5 cells transfected with sh*NC* or sh*ATOH1* were subcutaneously into the nude mice. Thioridazine-treated mice received 10 mg/kg of THO (HY-B0965, MCE, dissolved in sterile DMSO at 2 mg/mL and sterile filtered) via intraperitoneal injection every 3 days for 3 weeks (7 doses total) or until humane endpoint criteria were met. Control mice received an equal volume of sterile DMSO in the same manner. Mice were monitored regularly for health status and tumor size.

To evaluate the effect of 5-Azacytidine (5-AzaC) on tumor growth, 2×10^6 of indicated AGS or SNU-5 cells were subcutaneously into the nude mice. When tumors became visible (~3 × 3 mm in size, approximately 7 days), the mice were randomly divided into two groups of five animals and treated intraperitoneally with 5-AzaC

(HY-10586, MCE, Monmouth Junction, NJ, USA) at a dose of 0.5 mg/kg body weight every other day for 3 weeks, whereas the control group was treated with an equivalent volume of DMSO. At the end of the treatment, the animals were sacrificed.

Mouse xenograft tumor treatment model

First, a xenograft tumor was induced by subcutaneously injecting 2×10^6 of AGS cells in 4-6 weeks of age female BALB/c nude mice. When the tumor volume reached >1cm^3 (about 4 weeks), the tumor was removed and cut into ~1mm^3 pieces and subcutaneously implanted into 4-6 weeks of age male nude mice. When tumors reached approximately 5 mm in diameter, the nude mice were divided into 4 groups (6 mice per group). Group 1 received an intratumoral injection of LV-Vector at a dose of 2×10^7 MOI in 20μL of PBS once per week for 4 weeks. Group 2 received an intratumoral injection of LV-*ATOH1* at a dose of 2×10^7 MOI in 20μL of PBS once per week for 4 weeks. Group 3 received an intratumoral injection of LV-Vector once per week for 4 weeks, plus an intraperitoneal injection of 5-FU (40mg/kg body weight) twice per week for 3 weeks. Group 4 received an intratumoral injection of LV-*ATOH1* once per week for 4 weeks, plus an intraperitoneal injection of 5-FU (40mg/kg body weight) twice per week for 3 weeks.

THO treatment

Mice were intraperitoneally injected with 10 mg/kg THO (HY-B0965, MCE, Monmouth Junction, NJ, USA) dissolved in PBS.

5-FU treatment

Mice were intraperitoneally injected with 50 mg/kg 5-FU (HY-90006, MCE, Monmouth Junction, NJ, USA), dissolved in PBS, either alone or in combination with 10 mg/kg THO.

REFERENCE

[1] Q. Y. Chen, Q. Zhong, Z. Y. Liu, J. W. Xie, J. B. Wang, J. X. Lin, J. Lu, L. L. Cao, M. Lin, R. H. Tu, Z. N. Huang, J. L. Lin, P. Li, C. H. Zheng, C. M. Huang, *Annals of surgical oncology* **2019**, *26* (6), 1759, https://doi.org/10.1245/s10434-019-07217-x.

[2] M. D'Angelica, M. Gonen, M. F. Brennan, A. D. Turnbull, M. Bains, M. S. Karpeh, *Ann Surg* **2004**, *240* (5), 808, https://doi.org/10.1097/01.sla.0000143245.28656.15.

[3] J. B. Wang, P. Li, X. L. Liu, Q. L. Zheng, Y. B. Ma, Y. J. Zhao, J. W. Xie, J. X. Lin, J. Lu, Q. Y. Chen, L. L. Cao, M. Lin, L. C. Liu, N. Z. Lian, Y. H. Yang, C. M. Huang, C. H. Zheng, *Nature communications* **2020**, *11* (1), 6352, https://doi.org/10.1038/s41467-020-20260-7.

[4] V. Kumar, K. Ramnarayanan, R. Sundar, N. Padmanabhan, S. Srivastava, M. Koiwa, T.

Yasuda, V. Koh, K. K. Huang, S. T. Tay, S. W. T. Ho, A. L. K. Tan, T. Ishimoto, G. Kim, A. Shabbir, Q. Chen, B. Zhang, S. Xu, K. P. Lam, H. Y. J. Lum, M. Teh, W. P. Yong, J. B. Y. So, P. Tan, *Cancer discovery* **2022**, *12* (3), 670, https://doi.org/10.1158/2159-8290.Cd-21-0683.

[5] a) A. Sathe, S. M. Grimes, B. T. Lau, J. Chen, C. Suarez, R. J. Huang, G. Poultsides, H. P. Ji, *Clinical cancer research : an official journal of the American Association for Cancer Research* **2020**, *26* (11), 2640, https://doi.org/10.1158/1078-0432.Ccr-19-3231; b) P. Zhang, M. Yang, Y. Zhang, S. Xiao, X. Lai, A. Tan, S. Du, S. Li, *Cell reports* **2019**, *27* (6), 1934, https://doi.org/10.1016/j.celrep.2019.04.052.

[6] G. Yu, L. G. Wang, Y. Han, Q. Y. He, *Omics : a journal of integrative biology* **2012**, *16* (5), 284, https://doi.org/10.1089/omi.2011.0118.

[7] H. Lian, Y. P. Han, Y. C. Zhang, Y. Zhao, S. Yan, Q. F. Li, B. C. Wang, J. J. Wang, W. Meng, J. Yang, Q. H. Wang, W. W. Mao, J. Ma, *Mol Oncol* **2019**, *13* (10), 2227, https://doi.org/10.1002/1878-0261.12557.

[8] D. Maeser, R. F. Gruener, R. S. Huang, *Briefings in bioinformatics* **2021**, *22* (6), https://doi.org/10.1093/bib/bbab260.

[9] a) R. Cristescu, J. Lee, M. Nebozhyn, K. M. Kim, J. C. Ting, S. S. Wong, J. Liu, Y. G. Yue, J. Wang, K. Yu, X. S. Ye, I. G. Do, S. Liu, L. Gong, J. Fu, J. G. Jin, M. G. Choi, T. S. Sohn, J. H. Lee, J. M. Bae, S. T. Kim, S. H. Park, I. Sohn, S. H. Jung, P. Tan, R. Chen, J. Hardwick, W. K. Kang, M. Ayers, D. Hongyue, C. Reinhard, A. Loboda, S. Kim, A. Aggarwal, *Nature medicine* **2015**, *21* (5), 449, https://doi.org/10.1038/nm.3850; b) S. C. Oh, B. H. Sohn, J. H. Cheong, S. B. Kim, J. E. Lee, K. C. Park, S. H. Lee, J. L. Park, Y. Y. Park, H. S. Lee, H. J. Jang, E. S. Park, S. C. Kim, J. Heo, I. S. Chu, Y. J. Jang, Y. J. Mok, W. Jung, B. H. Kim, A.

Kim, J. Y. Cho, J. Y. Lim, Y. Hayashi, S. Song, E. Elimova, J. S. Estralla, J. H. Lee, M. S. Bhutani, Y. Lu, W. Liu, J. Lee, W. K. Kang, S. Kim, S. H. Noh, G. B. Mills, S. Y. Kim, J. A. Ajani, J. S. Lee, *Nature Communications* **2018**, *9* (1), 1777, https://doi.org/10.1038/s41467-018-04179-8.

[10] L. Cao, S. Zhu, H. Lu, M. Soutto, N. Bhat, Z. Chen, D. Peng, J. Lin, J. Lu, P. Li, C. Zheng, C. Huang, W. El-Rifai, *Gastroenterology* **2022**, *162* (6), 1716, https://doi.org/10.1053/j.gastro.2022.01.046.
